# Harnessing Metabolomics to Advance Nutrition-Based Therapeutics for Inflammation: A Systematic Review of Randomized Clinical Trials

**DOI:** 10.3390/metabo15110705

**Published:** 2025-10-29

**Authors:** Belén Carlino, Gerardo N. Guerrero-Flores, Camila Niclis, Gina Segovia-Siapco, Martín L. Mayta

**Affiliations:** 1 Centro Interdisciplinario de Investigaciones en Ciencias de la Salud y del Comportamiento (CIICSAC), Facultad de Ciencias de la Salud, Universidad Adventista del Plata (UAP), Libertador San Martín 3103, Argentina; 2 Facultad de Ciencias Médicas, Universidad Nacional de Rosario (UNR), Rosario 2000, Argentina; 3 Instituto de Investigaciones en Ciencias de la Salud (INICSA)-CONICET-Universidad Nacional de Córdoba (UNC), Córdoba 5000, Argentina; cniclis@fcm.unc.edu.ar; 4 Center for Nutrition, Healthy Lifestyle, and Disease Prevention, School of Public Health, Loma Linda University, Loma Linda, CA 92350, USA; 5 Facultad de Ciencias Bioquímicas y Farmacéuticas, Universidad Nacional de Rosario (UNR), Rosario 2000, Argentina

**Keywords:** metabolomics, diet, inflammation

## Abstract

Background/Objectives: The association between plasma metabolites derived from dietary substrates and inflammatory processes remains underexplored, despite its potential relevance in the prevention of non-communicable diseases. This systematic review aimed to examine the relationship between blood metabolites and the modulation of inflammatory biomarkers. Methods: A total of 25 randomized controlled trials, published between 2019 and 2024, were included from an initial pool of 111 records. These studies investigated the effects of dietary patterns, specific food groups, or nutritional supplements on the human metabolome and their potential links to inflammation. Results: Metabolomic analyses were predominantly performed using mass spectrometry (MS)-based platforms (17 out of 25), with liquid chromatography–mass spectrometry as the most frequently employed method. Both targeted (*n* = 14) and untargeted (*n* = 11) approaches were represented, and samples were drawn from plasma, urine, and feces. Across the interventions, 64 metabolites were modulated, including fatty acyls, glycerolipids, benzenoids, and organic acids, reflecting potential changes in pathways related to oxidative stress, lipid and carbohydrate metabolism, and inflammatory signaling. Several studies also assessed classical inflammatory biomarkers such as C-reactive protein (CRP), tumor necrosis factor alpha (TNFα), interleukin-6 (IL-6), and monocyte chemoattractant protein-1 (MCP-1). Interventions involving healthy traditional dietary patterns, improvements in dietary fat quality, or the use of specific probiotic strains were often associated with favorable immunometabolic outcomes. In contrast, some interventions, such as Mohana Choorna, elicited upregulation of immune-related gene expression in adipose tissue without improvements in glucose or lipid metabolism. Conclusions: While metabolomic responses varied across studies, the evidence highlights the value of dietary interventions in modulating systemic metabolism and inflammation. These findings support the integration of metabolomics into clinical nutrition to define more personalized and effective dietary strategies for inflammation-related chronic disease prevention.

## 1. Introduction

Metabolomics is the study of the metabolome, the complete set of small molecules produced by cells [1,2]. Metabolomic data enable the correlation of metabolite profiles with phenotypes, known as metabotypes [3,4]. These profiles can fluctuate in response to genetic variation [5], environmental exposures [6], and lifestyle factors [7,8], all of which interact to influence health outcomes. Among these, diet is widely recognized as one of the most influential lifestyle factors [9]. Therefore, the characterization of small molecules derived from different dietary exposures enables researchers to evaluate how diet-induced alterations in metabolic profiles contribute to interindividual variation in metabotypes [10,11].

The identification of biomarkers through high-throughput metabolomic techniques has become a valuable strategy for unraveling the complex, bidirectional relationships between diet, metabolism, and inflammation [12,13]. This information supports the development of targeted nutritional strategies aimed at modulating inflammation and improving health outcomes.

The two primary analytical platforms in nutritional metabolomics are mass spectrometry (MS) and nuclear magnetic resonance (NMR) spectroscopy. On the one hand, MS-based techniques provide high sensitivity and broad coverage but typically require a prior separation method, such as liquid chromatography (LC) or gas chromatography (GC), to manage sample complexity [14,15,16,17]. GC-MS is effective for volatile and thermally stable compounds, including organic acids and amino acids, but often requires prior chemical derivatization, which limits its utility for less volatile metabolites [18]. LC-MS is more versatile, enabling the analysis of both polar and non-polar compounds without derivatization, and is now the most widely used platform in metabolomics [19]. However, LC-MS can be affected by ion suppression, where co-eluting compounds interfere with analyte ionization and reduce signal intensity [20]. On the other hand, NMR spectroscopy provides detailed structural information based on the magnetic properties of atomic nuclei. It is accurate, reproducible, and non-destructive, requiring minimal sample preparation and allowing for the analysis of living systems [21,22]. Moreover, NMR can identify unknown compounds using spectral information, even without reference standards [15]. Its main limitations are lower sensitivity compared to MS, necessitating higher metabolite concentrations, and signal overlap in complex mixtures [23]. Given these characteristics, NMR is frequently used as a complementary technique, while MS-based approaches remain dominant in clinical metabolomics.

The main approaches commonly used in metabolomics are untargeted and targeted [24]. Untargeted metabolomics seeks to detect a broad array of metabolites without prior selection, enabling the discovery of unanticipated compounds and generating comprehensive metabolic “fingerprints” of nutritional states [25]. However, it also presents important challenges: (a) it produces large, complex datasets that require advanced data processing; (b) it is prone to false discoveries, (c) many detected features remain unidentified (the so-called “dark metabolome”) [26,27], and additionally, (d) lower-abundance metabolites may be masked by more abundant compounds [20]. In contrast, targeted metabolomics focuses on a predefined set of compounds, offering higher reproducibility and simpler data analysis, making it ideal for hypothesis-driven studies [28]. Thus, while untargeted approaches are more suited for biomarker discovery and pathway exploration, targeted approaches provide the possibility to quantify a set of well-characterized metabolites, often those with known biological importance. These two strategies are therefore complementary and have been combined to yield broader insights [18,29].

Advances in these metabolomic platforms have made them essential tools for assessing individual health status [30]. As thousands of metabolites in human plasma reflect complex interactions between genetic, environmental, and lifestyle factors [31], metabolomic profiling is increasingly used to identify novel biomarkers for monitoring the impact of medical treatments and lifestyle changes. This is especially relevant in the context of chronic non-communicable diseases, such as diabetes, cardiovascular diseases, and cancer, which are now the leading causes of death worldwide [32].

A common underlying factor in the development of these diseases is chronic low-grade inflammation [33], characterized by persistently elevated levels of pro-inflammatory mediators and circulating immune cells [34,35,36]. Although this inflammatory state may not immediately damage tissues, it contributes significantly to disease progression [37]. Metabolomic analyses have identified specific molecules that could be involved in inflammation. These include metabolites that are significantly associated with both inflammatory and anti-inflammatory processes. This highlights their potential for use in prevention and therapeutic strategies [38].

Among the key factors influencing metabolism and the production of inflammation-modulating metabolites, diet plays a pivotal role [39]. Nutritional intake shapes both host and microbiota-derived metabolites, and diet-derived metabolites generated by the gut microbiota substantially affect host metabolism [40]. Diets high in saturated fatty acids (SFAs), for example, are associated with decreased microbial diversity and increased proliferation of pathogenic species [41]. This microbial imbalance damages the gut barrier, facilitating the translocation of bacterial components into the bloodstream, triggering systemic inflammation and promoting insulin resistance. In contrast, several diet-derived metabolites exert anti-inflammatory effects [42]. For instance, short-chain fatty acids (SCFAs), such as butyrate, produced by microbial fermentation of dietary fiber, have been shown to reduce inflammation, strengthen the gut barrier, and positively modulate the immune response [43,44,45].

Although numerous dietary intervention studies have explored these mechanisms, randomized controlled trials (RCTs) are considered the gold standard for establishing causal relationships. Unlike observational or cross-sectional designs, RCTs minimize bias, reduce confounding, and provide a temporal sequence between dietary exposures, metabolite responses, and inflammatory outcomes, making them essential to validate mechanistic pathways [46,47]. Yet, the role of host and gut-derived metabolites as mediators of inflammatory processes and the efficacy of targeted dietary interventions to modulate these pathways have not been sufficiently studied through RCTs. Moreover, despite clear evidence that diet and microbiota-derived metabolites modulate inflammation, the lack of long-term RCTs with mechanistic and translational approaches has led to fragmented knowledge. Addressing these causal relationships, while considering host–diet–microbiota interactions, is crucial to understanding the long-term metabolic effects of dietary patterns on gut health and inflammation.

While previous reviews have explored the effects of diet on inflammation or the human metabolome separately, few have comprehensively integrated evidence from randomized controlled trials examining both metabolomic responses and inflammatory biomarkers across diverse dietary interventions. This review addresses this gap by systematically synthesizing recent findings on how specific foods, dietary patterns, and supplements modulate metabolites linked to inflammation. By highlighting novel metabolite-biomarker associations and methodological innovations, this work advances understanding of the mechanistic links between diet and immunometabolic regulation and provides a foundation for more personalized nutritional strategies aimed at preventing inflammation-related chronic diseases.

In this context, a structured analysis of findings from randomized trials is needed to fill these knowledge gaps and provide a deeper understanding of how dietary intake influences metabolomic profiles and inflammation.

Accordingly, this systematic review aims to examine the impact of different dietary substrates on metabolomic signatures and how effects on metabolism modulate inflammatory responses.

## 2. Materials and Methods

The systematic review was conducted following the Preferred Reporting Items for Systematic Reviews and Meta-Analyses (PRISMA) checklist guidelines (Table S1) [48]. This review protocol was not registered on any platform. 

### 2.1. Search Strategy

An electronic search was performed using PubMed, Cochrane Library, and Epistemonikos databases (last searched in September 2024) to identify all articles related to the role of different dietary substrates on the profiles of metabolites present in humans and how they influence inflammatory tone. The following combination of Medical Subject Heading (MeSH) terms and text words were used: (Inflammation) AND (Nutrition OR Food OR Nutrient* OR Diet) AND (Metabolomic OR Metabolomics OR Metabolome OR Lipidomic OR Lipidomics OR Lipidome OR “Metabolic Profile” OR “Metabolic Profiles” OR “Metabolic Profiling” OR “Metabolite Profile” OR “Metabolite Profiles” OR “Metabolite Profiling”).

### 2.2. Eligibility Criteria

This study only included original publications written in English. Reviews and meta-analyses, comments, guidelines, editorials or letters, conference summaries, and non-randomized or non-controlled studies were excluded. The inclusion and exclusion criteria are outlined in Table 1.

### 2.3. Data Collection Process

Data was extracted in a standardized format into a Microsoft Excel^®^ spreadsheet. The following information was collected from each included study: Title, Authors, Year of Publication, Journal, DOI, Search Query, Date of Inclusion in the Database, Filters, RCT Research, Source, Metabolomic Technique, Samples, availability of the paper Free/Not Free, and Additional Comments.

A quantitative meta-analysis was not conducted due to several limiting factors. First, the majority of the included studies did not provide access to raw metabolomic data, preventing reanalysis or data harmonization. Second, substantial heterogeneity existed in the metabolites quantified and in the analytical platforms employed, including liquid chromatography–tandem mass spectrometry (LC–MS/MS), nuclear magnetic resonance (NMR), and other targeted or untargeted approaches. Additionally, differences in sample types, reporting formats, and normalization strategies further restricted data comparability across trials.

### 2.4. Screening and Selection

Four reviewers independently assessed and selected the studies based on the inclusion, exclusion, and quality assessment criteria outlined in the Risk of Bias Tool (Rob2). Discrepancies were resolved by discussion to reach a consensus.

### 2.5. Risk of Bias Assessment

The quality of the included studies was assessed using the Cochrane risk of bias tool for randomized trials (Rob2) (https://methods.cochrane.org/bias/resources/rob-2-revised-cochrane-risk-bias-tool-randomized-trials, accessed on 17 November 2024). The Rob2 tool allows for the assessment of bias that may arise at different stages of an RCT across five distinct domains. The five assessed domains represent: (1) bias in the randomization process; (2) bias due to deviation from intended interventions; (3) bias due to missing outcome data; (4) bias in the measurement of the outcome; and (5) bias in the selection and reporting of results. Each domain was rated as having a “low risk”, “some concerns”, or “high risk” of bias.

## 3. Results

### 3.1. Study Selection

**Figure 1:**
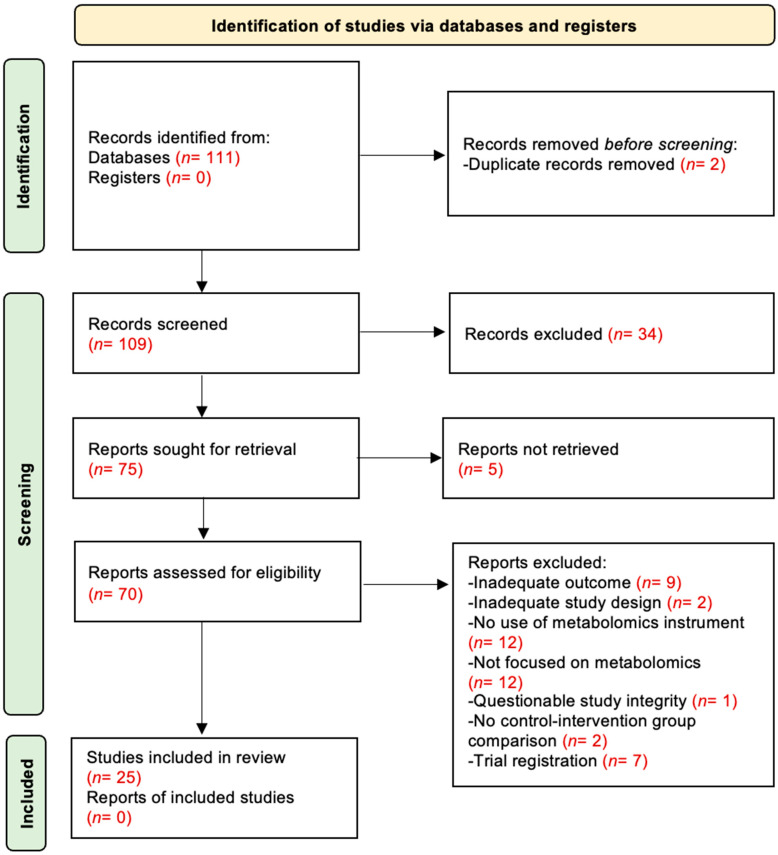
PRISMA flow chart illustrating the selection process of the studies.

The flow diagram of the study selection process is shown in Figure 1. In the original search, 111 records were published from 2019 to 2024. Before screening, 14 records were removed: 2 duplicates, 7 trial registrations, and 5 records with inaccessible full text. A total of 97 records remained and were screened by title and abstract. Of these, 34 were excluded for not meeting eligibility criteria. The remaining 63 articles were assessed in full-text form. Among those, 38 were excluded for the following reasons: inadequate outcome (*n* = 9), inadequate study design (*n* = 2), no conventional metabolomics instrument used (i.e., methods other than MS or NMR) (*n* = 12), not metabolomics-focused (studies centered on biomarkers rather than direct metabolite profiling) (*n* = 12), questionable study integrity (*n* = 1), and absence of a comparison group between intervention and control (*n* = 2). Ultimately, 25 studies met all inclusion criteria and were included in the systematic review. Of these, 8 were crossover RCTs and 17 were parallel-group RCTs.

### 3.2. Study Characteristics

The main characteristics of the included studies are summarized in Table S2, while comprehensive details are provided in Table S3. Ten studies were published in 2019 [49,50,51,52,53,54,55,56,57,58], three in 2020 [59,60,61], six in 2021 [62,63,64,65,66,67], one in 2023 [68], and five in 2024 [69,70,71,72,73]. The geographical origins of the studies were diverse. Most were conducted in Europe, particularly in Greece [62], Italy [51,62], Serbia [62], Spain [52,69,70], the United Kingdom [49,60], Netherlands [53,61,63], Denmark [55,58], Portugal [72], Finland [55], Sweden [54,55], Iceland [55], and Norway [56]. Three were conducted in the United States [64,65,70], two in Australia [67,71], and the rest in Asia: Iran [50], Korea [59,73,74], and China [57,66]. Of these, three were multi-centric: two international [55,62] and one intercontinental [70]. The sample sizes ranged from 10 [67] to 217 [57], with a total of 1654 individuals across all studies. The analysis included healthy individuals as well as those diagnosed with conditions such as non-alcoholic fatty liver disease, overweight or obesity, elevated blood pressure, impaired glucose tolerance, vitamin D deficiency, hypercholesterolemia, depressive symptomatology, rheumatoid arthritis, asthma, cirrhosis, and hypertension.

One study included only men [67], while four were conducted exclusively in women [52,53,54,59]. The remaining studies included participants of both sexes, except for one [68] that did not report on sex. The mean age of the participants ranged between 41 [73] and 81 [51] years. The duration of the intervention varied from 7 days [53] to 2 years [70].

Eight of the interventions focused on food or dietary patterns, such as the Mediterranean diet or dietary fat control. Fourteen involved supplementations, such as vitamin D and Korean red ginseng, and three involved probiotics (Table 2).

### 3.3. Analytical and Biological Heterogeneity

The selected studies show substantial methodological variability in crucial aspects such as: (1) sample type and preparation approaches, (2) choice of analytical platform, instrumentation, and optimization details, (3) calibration practices, and (4) reporting (or lack of) detection limits, all of which can affect comparability between the studies.

Regarding sample type, the most used was plasma (eight studies), followed by serum (five studies) and urine (four studies). A few studies included stool, muscle biopsies, or erythrocyte membrane fractions. Sample preparation strategies varied greatly between studies, as they are highly dependent on the analytical platform of choice (NMR or MS). A trend was evident regarding the choice of analytical platform, with most of the studies using MS (17/25) compared to NMR (6/25). Moreover, most studies (15/25) used LC as the prior separation technique. Among those, the most reported LC method was UPLC (13/15) with a C18 reverse phase column (10/15), and the most utilized detection system for MS was triple quadrupole (QQQ) (10/15) with electrospray ionization (ESI) (12/15). One study employed both GC-MS and LC-MS, while another study utilized GC-MS alone. Two studies used a combination of NMR and LC-MS to maximize metabolome coverage. The remaining study used GC but coupled with flame ionization instead of MS for metabolite detection (Table S2).

Targeted approaches (14/25) were slightly more common than untargeted approaches (10/25) in the analyzed studies. One study reported both targeted and untargeted approaches. However, no clear bias towards one type was observed. Differences instead reflected a choice based on whether there is a prior hypothesis being tested involving a given set of metabolites or not.

The dominant approach for sample preparation in MS-based studies was protein precipitation with an organic solvent, generally acetonitrile or methanol (15/17). Solid-phase extraction (SPE) with commercial cartridges was used in four studies. The three studies using GC-MS typically employed a two-step derivatization, but reagents and conditions were not uniform across these interventions. For NMR analysis, all studies reporting sample preparation used a similar and simpler approach consisting of the addition of an inorganic buffered solution prepared in deuterated water (D_2_O) for proton exchange (Table S2).

Regarding calibration practices, fourteen studies used relative quantification and seven employed absolute quantification using multi-point calibration curves or authentic standards. Moreover, 10 studies explicitly reported using an internal standard (spiked-in during sample preparation) for calibration. Most studies (11/25) used statistical methods for quality control (QC) of the data. However, analytical QC practices, such as the use of pooled QC samples, run throughout batches to monitor instrument stability and drift, which are essential to ensure proper data quality, are reported much less frequently (4/25). Four studies did not report any calibration practices. Almost all studies (23/25) did not report limits of detection for the detected metabolites (Table S2).

Among the 25 studies included, plasma was the most analyzed sample type, being used in 14 studies (56%). Serum samples were analyzed in 8 studies (32%), and urine samples in 5 studies (20%). Red blood cell (RBC) membranes and feces were each analyzed in 2 studies (8%). White blood cells and muscle biopsy specimens were the least commonly analyzed, each appearing in only 1 study (4%) (Table S2). These findings highlight a predominant focus on plasma and serum in the current literature, while other biological matrices remain underrepresented.

### 3.4. Metabolic Biomarkers Associated with Diet

**Figure 2:**
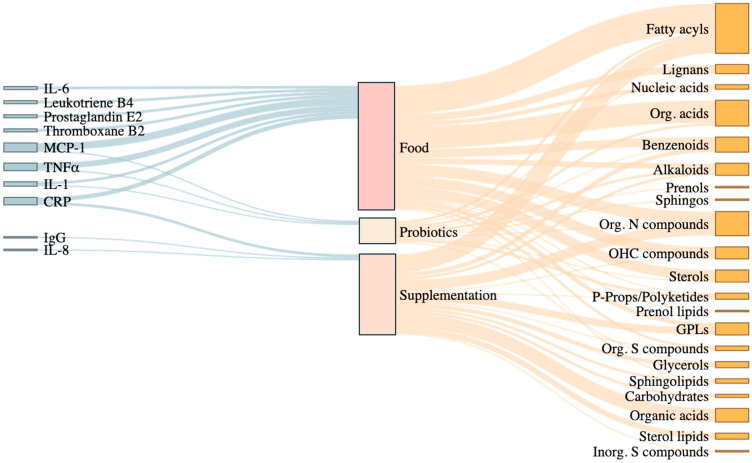
Sankey diagram illustrates the relationships between inflammatory biomarkers, dietary intervention types, and affected metabolite classes in the reviewed RCTs. The diagram shows the connections between key inflammatory biomarkers (e.g., IL-6, CRP, TNFα), the type of dietary interventions applied (Food, Supplementation, Probiotics), and the metabolite classes modulated in response. Food-based interventions are broadly associated with fatty acyls, organic acids, and nitrogen compounds, while supplementation and probiotics modulate particular classes of metabolites such as glycerophospholipids, benzenoids, and sphingolipids. The width of each flow reflects the frequency of associations reported across the reviewed studies, highlighting which metabolite classes are most consistently linked to dietary patterns and inflammatory outcomes. Org. Acids: organic acids; Sphingos: sphingolipids; Org. N compounds: organic nitrogen compounds; OHC compounds: organoheterocyclic compounds; P-props/Polyketides: phenylpropanoids and polyketides; GPLs: glycerophospholipids; Org. S compounds: organic sulfur compounds; Inorg. S compounds: inorganic sulfur compounds. Figure created using R (version 4.4.1) within RStudio (version 2024.09.0+375).

Table 3 shows the dietary interventions carried out in each study and the associated metabolites that showed either increases or decreases in responses, and Figure 2 represents this graphically. In every intervention, the type of sample analyzed is explained, and the metabolites are grouped into families. The RefMet classification [75] provided by Metabolomics Workbench (https://www.metabolomicsworkbench.org/, accessed on 1 February 2025), an international repository for metabolomics data and metadata, was used to place each metabolite according to the highest hierarchical level of classification of a metabolite (superclass). From this point forward, consensus on the classifications used will be based on those extracted from this source instead of those presented in each article. Metabolites that could not be classified in this source were assigned to a superclass using the Human Metabolome Database (HMDB) [76].

A total of 64 metabolites of 17 metabolite superclasses were found to be modulated by different dietary interventions: alkaloids, benzenoids, carbohydrates, fatty acyls, glycerolipids, glycerophospholipids, lignans, nucleic acids, organic acids, organic nitrogen compounds, organic oxygen compounds, organic heterocyclic compounds, organosulfur compound, phenylpropanoids and polyketides, prenol lipids, sphingolipids, sulfur inorganic compounds, and sterol lipids.

Diets targeting oxidative stress have been shown to increase amino acids such as leucine, methionine, and glutamine, as well as allantoin, while decreasing fatty acyls such as oleic acid and nitrogen compounds such as trimethylamine N-oxide. Interventions targeting microbiota metabolism showed an increase in long-chain fatty acids, such as docosahexaenoic acid, and also essential amino acids, while reducing organic nitrogen compounds, such as 8-oxo-2’-deoxyguanosine. Similarly, diets targeting lipid metabolism were associated with increased polyunsaturated fatty acids (PUFAs). On the other hand, those diets targeting carbohydrate metabolism increased oxidized low-density lipoprotein (ox-LDL) and glutamate, while reducing oleic acid, pseudouridine, and nitrogen-containing compounds such as choline. Interventions aimed to improve inflammatory biomarkers also increased ox-LDL, and essential amino acids, while reducing lysophosphatidylcholines (a type of glycerophospholipid) and oxidative DNA markers [78] like Oxo-2’-deoxyguanosine and N-acetylglycoproteins [69].

Other general dietary interventions targeting specific cultural or national patterns were associated with changes in key metabolic pathways. For instance, the typical Western diet observed in the U.S. population was associated with an increased presence of benzenoids, such as hippuric acid, fatty acyls, organoheterocyclic compounds, organic acids, and steroid lipids, while reducing the presence of lignans, such as enterodiol-glucuronide [73]. In contrast, the Healthy Nordic Diet, characterized by a high intake of whole grains, canola oil, berries, and fish, showed increased levels of pipecolic acid, betaine, and nitrogen compounds [55]. The balanced Korean diet was associated with increased benzenoids, fatty acyls, lignans, sphingolipids, and sterol lipids. With a similar metabolomic profile, the 2010 Dietary Guidelines for Americans promoted an increase in organoheterocyclic compounds, like creatinine [73].

Dietary interventions that modify nutrient intake or the consumption of specific food groups were associated with distinct changes in metabolite profiles across various biological matrices. For example, walnut-enriched diets have been shown to increase oxylipins like 5,6-DiHETE and 5-HETE, while reducing fatty acyls such as 9-HOTrE and 12,13Epoxy9(E)octadecenoic acid. The addition of cruciferous vegetables to the diet increases S-methyl cysteine sulfoxide in urine, while reducing it in plasma and serum carotenoids (prenol lipids) [70]. Whole grain-rich diets, on the other hand, lead to an increase in urine metabolites such as benzenoids and fatty acyls, as well as organic acids like 2-aminophenol sulfate [58]. Replacing SFAs with PUFAs in the diet increases short chain fatty acids such as acetate, water-soluble vitamin such as thiamine, and organic acids such as serine and proline in plasma, while reducing lipoproteins, cholesterol, and other fatty acids such as palmitoylcarnitine [56]. Moreover, fat-controlled diets changed the levels of different alkaloids, such as indole and indoleacetic acid, and fatty acids, such as palmitic acid, stearic acid, and arachidonic acid, in fecal samples [57].

Supplementation interventions revealed several notable metabolomic shifts. In particular, many of the studies reported increases in specific fatty acids and lipids. For example, high-dose vitamin D3 supplementation increased phosphatidylcholine and sphingomyelin. Moreover, the effect of vitamin D3 supplementation doses on glycerolipids varied depending on the dose. While the low dose tended to decrease 40:4 diglycerol, the high dose increased it and decreased 36:3 [53]. Eicosapentaenoic acid supplementation increased and decreased different types of PUFAs [65]. Cholecalciferol and blue mussel supplementation led to a reduction in some types of carbohydrates, such as glucose. Additionally, with cholecalciferol supplementation, certain organic acids (aminoacids) were consistently modulated. For example, the levels of glutamine and histidine increased [72], whereas *Bifidobacterium lactis* Probio-M8 combined with Symbicort Turbuhaler supplementation increased the levels of succinic acid, L-tryptophan, and curcumin–phospholipid complex, resulting in decreased levels of various metabolites belonging to the superclass of organic acids [66].

Although less commonly reported, some interventions affected specific metabolite subclasses such as organic oxygen compounds, benzenoids, phenylpropanoids and polyketides. For instance, supplementation with curcumin–phospholipid complex showed a decrease in kynurenine [50], and the probiotics *Bifidobacterium bifidum* BGN4 and *Bifidobacterium longum* BORI showed a decrease in indole-3-acetic acid [74]. In addition to probiotic interventions, supplementation with Korean red ginseng [57], curcumin–phospholipid complex [50], and shark liver oil [67] decreased sterol lipids. Supplementation with liquid essential amino acids combined with whey protein and vitamin D [51] increased an organic oxygen compound (N-acetyl-L-leucine), while supplementation with xanthophylls, anthocyanins combined with xanthophylls [52], nicotinamide riboside [61], and *Bifidobacterium lactis* Probio-M8 powder combined with Symbicort Turbuhaler, a nasal inhaler used for asthma prevention, decreased organic nitrogen compounds [66]. Conversely, benzenoids like phenol sulfate increased in diet supplemented with anthocyanins and xanthophylls [52], strawberry powder [64], and *Bifidobacterium lactis* Probio-M8 powder combined with Symbicort Turbuhaler [66], and decreased with curcumin–phospholipid complex consumption [50]. Phenylpropanoids and polyketides were only present when urolithin A increased with strawberry supplementation [64]. Prenol lipids only decreased with cruciferous vegetables servings added to diet [71].

### 3.5. Dietary Interventions and Their Effects on Inflammation

Table 3 also shows the inflammatory biomarkers measured in each study. The most frequently assessed biomarkers across the studies were C-reactive protein (CRP), tumor necrosis factor-alpha (TNFα), monocyte chemoattractant protein-1 (MCP-1), and various interleukins (IL-6, IL-1β, and IL-8).

Personalized diets designed to optimize carbohydrate and oxidative stress biomarkers were linked to higher TNFα and MCP-1 levels than a Mediterranean diet-based control, while diets focused on lipid metabolism were associated with a reduction in CRP but an increase in TNFα [69]. Additionally, some studies explored genetic biomarkers related to inflammation and lipid metabolism [56,63]. In one case, during the Mohana Choorna supplementation, where SFAs were replaced with PUFAs, the expression of genes involved in inflammation was upregulated [63].

The balanced Korean diet, the diets recommended by the 2010 Dietary Guidelines for Americans, and even the typical U.S. diet, all demonstrated consistent decreases in MCP-1 levels [73]. Additionally, the healthy Nordic diet was found to reduce IL-1 through negative associations with pipecolic acid, betaine, and trigonelline [55]. Depending on the specific fat content, fat-controlled diets also showed reductions in CRP, thromboxane B2, prostaglandin E2, and leukotriene B4, while whole grain-rich diets were associated with decreased fasting serum IL-6, IL-1β, and TNFα [57]. Eicosapentaenoic acid and shark liver oil supplementation both showed a decrease in CRP levels [65]. On the other hand, some interventions showed no clear effects on classical inflammatory markers. For example, personalized diets targeting inflammation-related and microbiota metabolism biomarkers [69], addition of cruciferous vegetables [71], inulin [49], strawberries [64], nicotinamide riboside [61], and supplements rich in anthocyanins or xanthophylls [52], did not significantly change key markers such as IL-6, CRP, or Apo levels. However, probiotic interventions using strains like *Bifidobacterium bifidum* BGN4, *Bifidobacterium longum* BORI [68], and *Lactobacillus casei Shirota* [60] were associated with decreased MCP-1 levels.

### 3.6. The Impact on the Gut Microbiota

Data on gut microbiota were only obtained in some of the dietary interventions. Lower-fat diets have been associated with increased relative abundances of the genera *Blautia* and *Faecalibacterium*. In contrast, higher-fat diets tend to reduce *Faecalibacterium*, while increasing the abundance of *Alistipes* and *Bacteroides* [57].

Whole grain diets do not significantly alter overall bacterial diversity, but they do promote the growth of specific strains such as *Faecalibacterium prausnitzii* and *Prevotella copri*, while reducing *Bacteroides thetaiotaomicron* [58]. Inulin and inulin-propionate ester supplementation appear to decrease microbial diversity while selectively enriching certain beneficial taxa, including *Bifidobacterium faecale*, *Anaerostipes hadrus*, and *Fusicatenibacter saccharivorans*, while reducing others, such as *Blautia obeum* and *Prevotella copri* [49].

Probiotic interventions demonstrated taxon-specific shifts accompanied by changes in metabolite profiles. *Bifidobacterium bifidum* BGN4 and *Bifidobacterium longum* BORI increased *indole-3-propionic acid*, a tryptophan-derived microbial metabolite associated with reduced IL-1β and TNFα levels [74]. *B. lactis* Probio-M8 promoted the growth of *Bifidobacterium animalis* and *Roseburia hominis* alongside increased sphingomyelin and tryptophan derivatives [66].

Mastiha supplementation showed an increase in the Bray–Curtis microbiota dissimilarity index, indicating broader shifts in microbial community structure, alongside a reduction in *Flavonifractor* abundance [62].

## 4. Discussion

### 4.1. Metabolites Derived from Dietary Intervention in RCTs and Their Association with Inflammation

Integrating targeted and untargeted metabolomics into dietary intervention research provides a rigorous framework for identifying molecular mediators underlying the health effects of specific dietary patterns and supplements [79]. Rather than relying solely on clinical endpoints, combining both approaches allows to identify bioactive compounds, host-microbiota co-metabolites, and signaling molecules that reflect real-time biological responses to nutritional exposure [80].

Across the reviewed studies, diverse interventions ranging from whole dietary patterns to targeted supplementation, consistently modulated the levels of various metabolites and showed potential links to inflammatory biomarkers.

### 4.2. The Impact of a Whole-Food Diet on Metabolite Expression

Dietary patterns centered on whole, minimally processed foods, abundant in plants and healthy fats, sufficient lean and plant-based proteins, and limited alcohol, red meat, refined and ultra-processed items, induced consistent changes in metabolites associated with lipid metabolism [81], immune signaling, and microbial fermentation [55,58,69,73,82]. In Nordic and wholegrain diets, there are increases in trigonelline, pyrocatechol sulfate, and DHPPA-glucuronide, a phenylpropanoid derived from microbial processing of plant polyphenols and fibers. This bacterial activity promotes anti-inflammatory enterocytic communication [83]. Meanwhile, trigonelline, is recognized for its antioxidant and anti-inflammatory properties, as well as its role in lipid metabolism [84]. On the other hand, elevated levels of hippuric acid, vanillic acid 4-o-sulfate, and isobutyryl carnitine in a balanced Korean diet and the 2010 Dietary Guidelines for Americans have shown an increased β-oxidation and benzenoid processing, which may influence immune responses, either by contributing the resolution of inflammation or, conversely, by exacerbating it, depending on the context [85,86,87]. The increase in organoheterocyclic compounds, such as creatinine, associated with the 2010 Dietary Guidelines for Americans and unlike the healthy Korean diet, could be explained by its higher proportion of creatine-rich foods, such as lean meats, fish, and eggs, whose metabolism and cooking favor the generation of these metabolites [88]. 

Metabolic shifts promoted by healthy dietary types were associated with reductions in inflammatory cytokines (Figure 2), particularly IL-6, IL-1β, MCP-1, and TNFα [55,69,73], highlighting the interaction between microbial/host-derived metabolites and the canonical inflammatory cascade mediated by whole grain-derived metabolites [89]. Interestingly, in healthy individuals following the healthy Korean, 2010 Dietary Guidelines for Americans, and personalized diets designed to improve markers of carbohydrate metabolism and biomarkers of oxidative stress, changes in MCP-1 levels aligned with alterations in lipid-related metabolites, specifically carnitine derivatives, sphingolipids, and sterol lipids such as n,n-dimethyl-safingol and 11β-hydroxyandrosterone-3-glucuronide, respectively. This pattern may imply that lipid-derived signaling molecules or bile-acid pathways may influence chemokine regulation [90], resulting in reduced inflammation under controlled dietary conditions [73].

Similarly, diets enriched in walnuts decreased the levels of oxylipins (e.g., 5,6-DiHETrE and 5-HETE), which are mediators involved in the resolution of inflammation [91], while also reducing pro-oxidative fatty acid derivatives [70]. While other inflammation markers were not directly evaluated, these results could suggest that the beneficial effects of walnuts on vascular and brain health are mediated, at least in part, by dietary modulation of oxylipins and by inhibition of the soluble epoxide hydrolase enzyme [92].

Dietary sulfur-containing compounds are widely distributed and highly enriched in cruciferous vegetables [93]. The consumption of cruciferous vegetables was associated with increased levels of sulphur-containing metabolites (e.g., s-methylcysteine sulfoxide and sulforaphane), which possess anti-inflammatory properties and may contribute to reduced risk of chronic diseases, such as colorectal cancer [94]. Alongside the presence of sulphur-containing metabolites, no association with IL-6 or CRP was observed. The results of this type of intervention emphasize that even including specific food components in the diet may reshape those metabolic pathways relevant to inflammatory processes. In consonance with these results, it has also been observed that plant- and whole foods-based dietary patterns consistently reduce inflammatory biomarkers, such as CRP, IL-6, and TNFα, while simultaneously decreasing oxidative stress biomarkers [95,96], which points to them as valid anti-inflammatory and antioxidant approaches for their use in clinical settings. Accordingly, recent metabolomic studies have identified specific molecules, such as trigonelline, DHPPA-glucuronide family metabolites, acylcarnitines/carnitines, sphingolipids, and steroid glucuronides, as potential mediators of anti-inflammatory effects. These molecules act through β-oxidation, benzenoid metabolism, sphingolipid, and bile acid signaling pathways. For example, trigonelline is inversely associated with dietary inflammatory potential, while sphingomyelins and bile acids are altered in inflammatory diets [97].

Interestingly, a significant reduction in serum carotenoids, such as lutein, lycopene, and α- and β-carotene, was documented when cruciferous vegetables were added to the diet [71], which may reflect differences in antioxidant composition between the interventions rather than a loss of nutritional benefits from sulfur-containing compounds from cruciferous vegetables [98].

### 4.3. The Impact of Fatty Acids on Metabolite Expression

The second group of food interventions, which focused specifically on the quality and quantity of dietary fats, revealed distinct metabolomic signatures. Specifically, the substitution of SFAs with PUFAs increased metabolites such as acetate, bile acids, citrate, cystathionine, and enzymes like PCSK9 [56], suggesting the activation of cholesterol efflux mechanisms and the LXRα-LDLR axis [99]. Concomitantly, there was a reduction in atherogenic lipoproteins, specifically, very low-density lipoprotein and low-density lipoprotein, cholesterol esters, carnitine derivatives, and lipoproteins, reflecting improvements in lipid metabolism and probably a reduction in lipid-induced inflammation [100]. However, it should be noted that specific inflammation markers were not measured. 

Similarly, diets with varying fat content (20–40% of energy) revealed that high-fat diets were associated with lower levels of microbial-derived metabolites, such as butyric and valeric acid [57]. These patterns suggest that both the quality and content of fat can influence host–microbiota interactions and downstream inflammatory signaling by altering lipid, amino acid, and SCFAs metabolism [101,102].

All of these findings are consistent with the previous literature emphasizing the metabolic and inflammatory consequences of modulating dietary fat [103]. For example, recent studies show that diets lower in saturated fat and richer in unsaturated fats are associated with improved lipid profiles and reduced inflammatory biomarkers, such CRP and IL-6, supporting the notion that fat quality plays a key role in metabolic and inflammatory regulation [104,105]. Similarly, it has been shown that replacing SFAs with PUFAs or monounsaturated fatty acids (MUFAs) lowers cardiovascular risk by improving lipid metabolism and reducing inflammation-related gene expression [106]. Low-fat diets also modulate gut microbiota-derived metabolites, such as butyric acid and indole derivatives, suggesting an attenuation of systemic inflammation [21]. The consistency of these findings across various dietary contexts highlights the sensitivity of the metabolome to manipulation of dietary fat consumption and its potential for understanding the effects of dietary interventions on inflammation.

### 4.4. The Impact of Supplementation on Metabolite Expression

Further nuances were added to the context by supplementation studies. Vitamin D supplementation induces metabolomic alterations that strongly suggest immunoinflammatory modulation. Low-dose vitamin D_3_ supplementation was found to reduce circulating sphingomyelins and ether-linked phosphatidylcholines/phosphatidylethanolamines, both of which are precursors of ceramides [107], which play a crucial role both in cellular signaling and inflammation [108]. Therefore, the observed increase in sphingomyelin turnover [53], may contribute to reduced postprandial inflammation [109]. In contrast, high-dose supplementation reversed these benefits [53], highlighting the importance of appropriate dosing [110,111]. Moreover, vitamin D3 alone decreased blood glucose and acetate while increasing glutamine and histidine [72], all metabolites known to support immune cell function and inversely correlated with inflammatory biomarkers [112,113]. When combined with essential amino acids and whey protein, this intervention also resulted in increased oleic, palmitic, and stearic acids and glutathione reductase, and decreased omega-6 PUFAs [51]. Altogether, these changes suggest an improved redox balance and reduced lipid-mediated inflammation [114]. 

While two of the three interventions with vitamin D3 did not directly measure inflammation, the metabolite profiles indicated the presence of compounds with known anti-inflammatory properties, such as glutamine and histidine. Also, evidence of sphingolipid remodeling, shifts in fatty acid composition, and increased antioxidant enzyme activity suggests modulation of eicosanoid-mediated responses [115]. These changes point to enhanced macrophage regulation, reduced endothelial activation, and potential reductions in CRP and TNFα following vitamin D3 supplementation [116].

Several supplementation interventions revealed metabolite profiles indicative of anti-inflammatory or pro-resolution of inflammation activity. Eicosapentaenoic acid supplementation, for example, was associated with increased levels of n-3 PUFAs (20:5, 22:5, and 22:6), as well as decreases in certain n-6 PUFAs. There was also an increase in specialized mediators of inflammation resolution, such as LXB4 [65]. This supports the role of eicosapentaenoic acid in pathways involved in the resolution of inflammation [117]. On the other side, shark liver oil supplementation altered glycerophospholipid subclasses and reduced CRP, suggesting potential benefits through remodeling of the lipidome [22]. 

Curcumin–phospholipid complex consumption, although not directly assessed for inflammatory markers, reduced levels of metabolites such as indoxyl sulfate and kynurenine, both commonly elevated in inflammatory and microbiota-related metabolic dysregulation [118]. This suggests an indirect modulation of inflammation through the gut-liver axis and mitochondrial metabolism. In contrast, consumption of inulin-propionate ester, compared to inulin or cellulose, elevated circulating propionate and significantly reduced IL-8 levels, aligning with propionate’s known immunoregulatory properties [119]. These findings highlight the value of metabolomics in detecting systemic metabolic shifts that conventional inflammatory markers may overlook.

By contrast, other interventions were associated with either neutral or potentially adverse inflammatory profiles. This was the case of Mohana Choorna consumption, which, despite prior preclinical evidence of metabolic benefits [63], induced the upregulation of numerous inflammation and immune-related genes in human adipose tissue which might point to early disruptions in tissue health and progression towards metabolic dysfunction [36], without improving glucose or lipid metabolism, as previously reported [63]. This highlights the need to validate positive evidence obtained with animal models in properly designed human studies.

Supplementation with nicotinamide riboside reduced plasma acetylcarnitine, but no significant changes were observed across a broad range of inflammatory cytokines [61], suggesting a limited systemic impact on inflammation under the conditions studied [120].

### 4.5. The Impact of Probiotics Supplementation on Metabolite Expression

The reviewed probiotic interventions reported distinct metabolomic signatures, suggesting diverse potential immunomodulatory roles, through microbiota-derived metabolites with recognized anti-inflammatory properties [66,68]. For example, *Bifidobacterium lactis* Probio-M8 supplementation increased the levels of metabolites involved in anti-inflammatory pathways and mucosal immune homeostasis, such as sphingomyelin, syringic acid, and tryptophan [66] while decreasing the levels of molecules linked to metabolic stress and inflammation, such as tetracosanoic acid and 3-methylglutarylcarnitine [121].

*Bifidobacterium bifidum* BGN4 and *Bifidobacterium longum* BORI led to a significant increase in some tryptophan-derived metabolites, such as indole-3-propionic acid, produced by gut bacteria. This metabolite has been shown to reduce IL-1β and TNFα levels [68], and to help maintain the intestinal barrier integrity [122]. Additionally, the observed increase in secondary bile acids may reflect enhanced microbial activity and modulation of the gut-liver axis [123]. In contrast, *Lactobacillus casei Shirota* did not induce detectable changes in the metabolome, but reduced MCP-1 levels [60], suggesting immune modulation via mechanisms not directly related to metabolome remodeling [109]. These findings underscore how shifts in tryptophan metabolism, indole derivatives, and lipid mediators induced by probiotics may underlie anti-inflammatory effects [124,125].

Taken together, the reviewed evidence suggests that dietary modulation of the gut microbiota may represent a key mechanism underlying metabolomic and inflammatory responses. Diets promoting the growth of *Faecalibacterium*, *Roseburia*, and *Bifidobacterium,* major producers of butyrate and other SCFAs, are typically associated with enhanced intestinal barrier function and reduced expression of pro-inflammatory cytokines [126]. Conversely, the increased abundance of *Alistipes* or *Flavonifractor* has been linked to the generation of oxidative metabolites and elevated LPS-mediated signaling, consistent with low-grade inflammation [127].

These interactions may also extend beyond SCFAs, as several trials reported shifts in tryptophan metabolism and sphingolipid pathways that coincide with microbial changes [128]. For instance, *Bifidobacterium*-based interventions increased circulating indole-3-propionic acid and sphingomyelin, both recognized for their anti-inflammatory and epithelial-protective roles [74,129]. This convergence of microbiota and metabolomic changes supports the concept of an integrated diet–microbiota–metabolite–inflammation axis, though the small number of RCTs combining multi-omic analyses precludes causal inference. Further mechanistic and longitudinal studies are warranted to clarify these relationships.

### 4.6. Nutrient-Derived Metabolites in the Regulation of Inflammation

When nutrients and food-derived compounds are ingested, they undergo metabolic processing that can lead to the accumulation of metabolites linked with chronic inflammation. Examples include ceramides [130], diacylglycerols (DAGs) [131], and quinone derivatives of polycyclic aromatic hydrocarbons (PAH-quinones) [132,133]. During normal cellular metabolism, dietary biomolecules are directed toward the Krebs cycle, the central hub of energy production. Within this cycle, substrates are broken down to generate electron carriers, NADH and FADH_2_, which subsequently fuel ATP synthesis through the electron transport chain [134]. In inflammatory contexts, however, diet-derived molecules such as saturated fatty acids disrupt these processes. For instance, saturated fats can be metabolized in macrophages into ceramides and palmitate, which activate immune receptors, including Toll-like receptors (TLR2/4) and NLRP3/6 inflammasomes. This promotes polarization toward pro-inflammatory M1 macrophages. Under these conditions, the Krebs cycle becomes disrupted at key nodes, leading to the accumulation of intermediates such as citrate, succinate, and itaconate [135]. These shifts drive metabolic reprogramming toward aerobic glycolysis and enhance the production of reactive oxygen species (ROS) [136], further triggering pro-inflammatory pathways that contribute to chronic disease [137].

**Figure 3:**
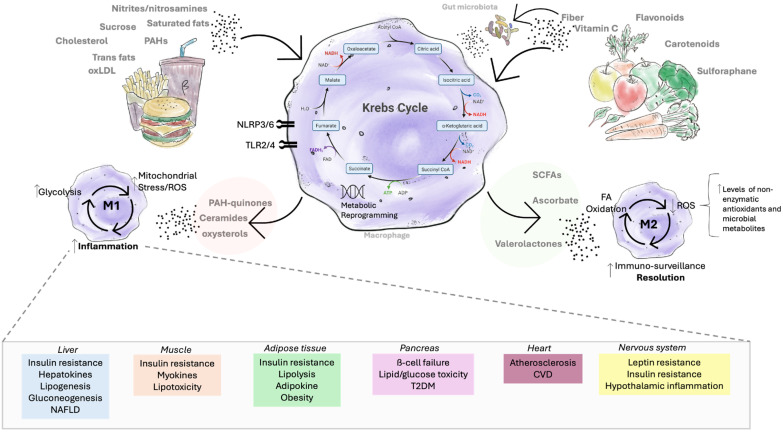
The effect of food metabolites on inflammation. Unhealthy lifestyle exposures such as high-palatable foods rich in trans fats, cholesterol, oxidized LDL (low-density lipoprotein), nitrites, nitrosamines, and polycyclic aromatic hydrocarbons (PAHs) activate innate immune receptors (e.g., TLR2/4, NLRP3/6) and drive metabolic reprogramming toward aerobic glycolysis, accumulation of Krebs cycle intermediates (e.g., citrate, succinate), mitochondrial stress, and augmented reactive oxygen species (ROS) production. For instance, metabolites derived from saturated fats (e.g., palmitic acid → ceramides) promote M1 macrophage polarization and chronic inflammation. This, in turn, contributes to systemic complications such as insulin resistance, steatosis, and non-alcoholic fatty liver disease (NAFLD) in the liver; myokine alterations and lipotoxicity in muscle; adipokine imbalance, obesity, and excessive lipolysis in adipose tissue; β-cell failure and type 2 diabetes in the pancreas; atherosclerosis and cardiovascular disease; and leptin resistance, insulin resistance, and hypothalamic inflammation in the nervous system. By contrast, healthy dietary compounds such as vitamin C and flavonoids promote fatty acid oxidation and enhanced antioxidant capacity (via non-enzymatic antioxidants: vitamin C → ascorbate; or via antioxidant microbial metabolites: catechins → valerolactones), thereby favoring M2 macrophage polarization, which favors immune resolution and tissue repair. Furthermore, fermentation of dietary fiber by gut microbiota releases short-chain fatty acids (SCFAs) (e.g., fiber → butyrate), while microbial catabolism of polyphenols produces phenolic metabolites (e.g., bound polyphenols → ferulic acid). Both classes of compounds reinforce M2 macrophage polarization and anti-inflammatory pathways.

The resulting inflammation impacts multiple organ systems, including the liver [138], muscle [139], adipose tissue [140], pancreas [141], heart [142], and the nervous system [39], thereby contributing to disease pathogenesis. By contrast, consumption of diets rich in fiber and phytochemicals (e.g., vitamins and flavonoids) supports polarization toward an M2 macrophage phenotype, associated with immune resolution and tissue repair [143]. For example, vitamin C, metabolized to ascorbate, can promote M2-like macrophage differentiation at appropriate levels, while catechins exert similar effects. Additionally, fermentation of dietary fiber by the gut microbiota yields short-chain fatty acids (SCFAs), which further favor M2 macrophage polarization [144] (Figure 3).

### 4.7. The Effects of Analytical and Biological Heterogeneity on the Comparability of Metabolomic Studies

The reviewed interventions showed important analytical variability, making comparability across studies more difficult. However, it is possible to observe a clear preference to use LC–MS-based approaches in the reviewed RCTs. This is in line with their higher sensitivity and versatility, as it can be used for both polar and non-polar compounds of a wide molecular mass range, making them highly popular in general for metabolomic studies [19]. Even though most interventions converged towards the use of LC–MS as their analytical platform, there was important heterogeneity regarding calibration practices, use of QC procedures, and reporting practices. This calls for a standardized checklist with minimum reporting requirements to be included in RCTs such as sample preparation details, full instrument details, acquisition parameters, calibration practices, and QC strategies [4,145]. This is crucial not only to improve reproducibility but to allow cross-study comparability [146]. Also, the limit of detection should be included, as the lack of this hinders the ability to determine whether a metabolite is absent or below the limit of detection, which makes it very difficult to assess the reliability of the reported findings [145]. Although reporting critical instrument parameters was slightly better than calibration practices, QC strategies, and detection limits in the reviewed studies, it was still inconsistent among them. Moreover, all studies using MS, even those using high-resolution methods, failed to transparently report practices on internal mass calibration, lock-mass correction, or mass accuracy performance. These are essential to demonstrate instrument stability and reliable mass assignments used for metabolite identification [20]. Researchers should also be encouraged to always share calibration curves and raw/processed data to improve transparency, reproducibility, and facilitate comparability of the results [147].

Metabolomic signatures are strongly matrix-dependent, which can limit the reliability, accuracy, and interpretation of results [148,149]. In the reviewed articles, plasma and serum samples were commonly used, as compared to urine, where high concentrations of salts, such as sodium chloride, and other compounds make metabolite detection more challenging. Urine’s matrix effect is problematic, often time-consuming for equipment cleaning, and prone to adduct formation [150]. Plasma and serum primarily reflect systemic lipid and amino acid metabolism [151,152,153], whereas urine captures short-term fluctuations in microbial- and diet-derived metabolites, particularly phenolic derivatives [154,155]. Fecal metabolomics, although less frequently applied, provides complementary insights into gut-derived metabolites that influence, for example, host inflammatory responses [156,157,158,159].

Importantly, researchers should consider not only matrix effects but also the biological distribution of the metabolite of interest, as different compounds are more likely to be detected in specific matrices. The choice of biological matrix plays a critical role in shaping metabolite–inflammation associations, as plasma, urine, and feces capture distinct metabolic processes and time scales of exposure [160,161,162]. Plasma often reflects short-term metabolic responses and circulating bioactive compounds [163,164], while urine primarily captures excreted metabolites and may provide a cumulative measure of dietary intake or microbial catabolism [165,166,167]. In contrast, fecal samples better represent gut microbial activity and local metabolite production, although their relationship to systemic inflammation is less direct [168,169]. This heterogeneity complicates comparisons across studies, as the same metabolite may show different associations depending on the matrix analyzed. Moreover, the interpretation of statistical versus biological significance remains an important challenge. While some studies report significant associations at the nanomolar level, such changes may not always translate into physiologically relevant anti-inflammatory effects [170,171]. Distinguishing meaningful biological signals from minor fluctuations is therefore essential to advance metabolomics toward clinical and translational applications.

These matrix-specific differences underscore the importance of standardized biospecimen selection and multi-matrix experimental designs to capture comprehensive metabolic pathway interactions. Consequently, biomarker validation in nutritional metabolomics requires harmonization across matrices to enhance comparability and reproducibility of findings.

### 4.8. Conflicting Findings and Possible Moderators

Although many interventions suggest anti-inflammatory metabolic effects, results across RCTs remain inconsistent. Some studies reported increases in pro-inflammatory or neutral metabolites (e.g., ox-LDL, specific fatty acids, kinases) without corresponding changes in CRP or IL-6 [52,69]. Such discrepancies may reflect differences in nutrient dose [172,173], intervention duration [174,175], baseline metabolic or microbiota profiles, and the biological matrix analyzed (e.g., plasma vs. feces) [176]. Moreover, individual variability in absorption and metabolism may also contribute [177,178].

Recent dose–response metabolomics studies show that metabolic effects generate a stagnation beyond certain points, suggesting nonlinear relationships between intake and biochemical response [164]. However, few RCTs explore this systematically, and some lack statistical power to detect changes in inflammatory biomarkers. Additionally, metabolomic shifts do not always translate into corresponding cytokine responses, suggesting, in some contexts, a potential uncoupling between metabolic and inflammatory outcomes [107].

Vitamin D-related interventions illustrate this heterogeneity. In one trial differing only by dose, phosphatidylcholines and sphingomyelins changed in opposite directions [53]; others showed increases in glucose, creatinine, and fatty acyls [72], or positive correlations between palmitic/stearic acids and CRP [51]. These divergent responses probably are the reflection of differences in formulation, dose, duration, or co-interventions [179].

Only three of the included trials explicitly tested different doses or dietary levels of the same intervention: (i) fat-controlled diets with varying proportions of energy from fat (20%, 30%, 40%) [57], (ii) vitamin D_3_ supplementation at low vs. high doses [53], and (iii) EPA supplementation at 1 g/day, 2 g/day, and 4 g/day [65]. These studies provide valuable insights but also underscore the complexity of dose–response relationships in metabolomics. For instance, while the low-fat diet (20% energy) increased protective metabolites such as butyric acid, the high-fat diet (40% energy) led to increases in arachidonic acid and indole derivatives [57], which are more closely linked to pro-inflammatory pathways [115].

The duration of dietary interventions is another crucial factor shaping metabolomic and inflammatory outcomes; in the analyzed clinical trials, intervention durations ranged from seven days to 24 months. Short-term RCTs (e.g., ≤4 weeks) may capture acute metabolic responses or transient shifts in intermediary metabolites [180], whereas long-term interventions (≥6 months) are more likely to reflect adaptive remodeling of lipid and amino acid metabolism, as well as stable anti-inflammatory effects [181]. This temporal variability can partly explain why some trials reported early metabolite changes without concurrent alterations in inflammatory biomarkers, while others observed delayed but sustained improvements over time [182]. Such heterogeneity, further influenced by participants’ baseline metabolic status [183], methodological variability in metabolomic platforms [184], and differences in the metabolites measured through targeted techniques, highlights the complexity of diet–metabolite–inflammation interactions and underscores the need for harmonized study designs and standardized metabolomic approaches to allow more reliable cross-trial comparisons.

Overall, these findings indicate that heterogeneity stems from a combination of dose, duration, baseline metabolic status, and methodological variability in metabolomic profiling. Future RCTs should incorporate multiple dosage arms, standardized metabolite quantification, and longer follow-up to clarify whether responses scale with dose or plateau beyond a threshold. Meta-regression of dose, duration, and participant characteristics could further define functional metabolic thresholds linked to clinically relevant anti-inflammatory effects.

### 4.9. Clinical Implications

The findings of this review highlight the clinical potential of integrating metabolomic profiling into dietary interventions aimed at modulating inflammation. The consistent identification of metabolite patterns associated with anti-inflammatory effects, including trigonelline, carnitines, sphingolipids, and microbial-derived compounds, suggests that specific dietary strategies can induce measurable biological changes well before conventional clinical markers would detect them. This positions metabolomics as a valuable early detection tool for evaluating dietary efficacy and tailoring personalized nutrition approaches in clinical practice.

While metabolomics offers considerable promise for early disease detection and personalized nutrition, its implementation in clinical practice remains challenging. High analytical costs, limited accessibility of specialized instrumentation, and the absence of standardized protocols currently restrict its routine clinical use [185,186]. Moreover, variability in sample processing, data normalization, and metabolite annotation complicates cross-laboratory comparability and clinical validation [187,188]. To move toward clinical translation, efforts should focus on cost reduction, the development of standardized workflows, and integration with existing clinical platforms [83,189]. Establishing shared databases and reference ranges for key metabolites will also be essential to enable consistent interpretation and facilitate the incorporation of metabolomic profiling into personalized dietary counseling and preventive medicine.

### 4.10. Strengths and Limitations

The clinical RCTs included in this systematic review exhibited several methodological strengths, for instance: (a) some studies had relatively large participant numbers [56,57,70], (b) implementation of robust intervention designs [56,57,70,71], including double-blind protocols, which improved internal validity and reduced bias, (c) long intervention periods [57], (d) rigorous adherence monitoring that enabled the detection of long-term metabolic adaptations [70,71], and (e) confirmatory analyses to support metabolomic findings, contributing to the reliability of the reported results [54,64]. Ultimately, the novelty of their objectives and the originality of the metabolic signatures reported added significant value to the field [59,63,68,71,73].

Several methodological limitations were consistently observed across the included studies, which may affect the strength of the evidence synthesized in this review: (a) several trials were affected by pandemic-related constraints, such as limited in-person contact and reliance on electronic participation, which might have impacted adherence and reporting [69,71]; (b) small sample sizes, limited ethnic representation, wide age ranges, and lack of personalized or representative diets reduced generalizability [50,54,60,62,63,65,72,73]; (c) incomplete dietary controls posed risks of unreported food intake or medication use, introducing confounding factors [56,59,65,72]; (d) short intervention durations, absence of follow-up, single-blind designs, and inconsistent handling of biological samples, all potentially affecting data quality [50,59,61,63,69,70,73]; and (e) the absence of complementary analyses like biopsies or a comprehensive lipidome profiling, use of diverse metabolomic techniques, limits the understanding of the underlying mechanisms associated with the metabolomic observed metabolomics changes.

Building on the factors discussed in Section 4.6, several methodological aspects further constrain the interpretation and comparability of results across RCTs. This systematic review explored a broad range of dietary exposures and metabolites measured by several techniques, and while the heterogeneity in terms of study population and endpoints of each RCT makes it difficult to generalize findings between studies, some consistent patterns can still be identified. Another important limitation relates to the lack of standardization in metabolite identification and nomenclature across RCTs. In our synthesis, we observed that individual studies often relied on different reference databases, and in many cases, the database used was not reported at all. This inconsistency complicates efforts to unify metabolite names and categories, particularly in untargeted metabolomics, where metabolite annotation is inherently challenging. Although we used RefMet (a reference nomenclature for metabolomics) [75] as the primary framework for classification, not all metabolites could be retrieved from this source, and we therefore complemented our classification using HMDB [76]. The absence of a single comprehensive and universally adopted resource makes it difficult to achieve full consistency when comparing across multiple trials. Nevertheless, we recommend that future studies systematically adopt reliable and standardized resources, such as RefMet, HMDB, or PubChem [180], when reporting metabolite identities, as this would substantially improve clarity, reproducibility, and comparability between clinical studies.

## 5. Conclusions

The reviewed evidence supports dietary recommendations centered on whole-food-based patterns and improved fat quality, such as Mediterranean, Nordic, and low saturated fat diets, for their capacity to favorably modulate metabolic and inflammatory pathways. Metabolomic findings strengthen the evidence linking these dietary approaches with whole-food-based and improved-fat-quality in shaping metabolites-health associations, even though not each intervention showed a consistent reduction in chronic inflammation. Importantly, the results highlight the value of incorporating multiomic analyses to unravel the complex interactions between diet, gut microbiota, metabolism, and immune responses.

For a clinical standpoint, the findings reinforce the potential of incorporating personalized dietary advice to promote immune balance trough nutrient-microbiota-host interactions. Presenting such connections, in clinically relevant terms, helps bridge the gap between molecular evidence and nutritional guidance, facilitating their interpretation and application in clinical practice.

Future research should prioritize integrative designs that combine metabolomics with conventional clinical endpoints, such as inflammatory and metabolic markers, in long-term randomized trials. Observational studies are also encouraged to include metabolomic assessments to capture real-world dietary exposures and interindividual variability to complement experimental evidence. In parallel, the adoption of standardized protocols, shared data repositories, and harmonized analytic pipelines will be essential to ensure reproducibility and comparability across studies, thereby advancing precision nutrition strategies tailored to individual metabolic profiles.

Together, these approaches will help translate metabolomic insights into effective, personalized dietary strategies for the prevention of chronic inflammation and related diseases.

## Figures and Tables

**Table 1:** Inclusion and exclusion criteria.

Category	Inclusion Criteria
Population	Subjects older than 19 and healthy.
Study design	Randomized controlled trials.
Intervention	Dietary supplementation, dietary replacement, personalized nutrition, and probiotic supplementation.
Comparator	Placebo: no probiotic group; no supplement group; usual diet with saturated fat; diet with no strawberries; refined grain diet; non-cruciferous vegetable diet; no Ginseng, etc.
Outcomes	Metabolic health, inflammatory indicators, and gut microbiome modulation.
Language of publication	English; Spanish.
Category	Exclusion criteria
Missing data	Studies that report incomplete values (as lacking uncertainty) when the authors could not provide this information when requested.
RCT = randomized controlled trials	

**Table 2:** Main characteristics of the randomized controlled trials analyzed, organized according to the type of dietary intervention.

Reference (Country)	*n*	Participants	Mean Age, yrs	Intervention/Control Groups (*n*)	Duration of Intervention	Main Results
Amerikanou, C., et al. (Greece, Italy, Serbia, 2021) [62]	98	Patients with NAFLD	48.83 ± 9.36	Mastiha supplementation (41)/Placebo (57)	6 months	In severely obese NAFLD patients, Mastiha reduced liver inflammation markers and decreased LysoPC 18:1, LysoPE 18:1, and cholic acid, alongside modulating gut microbiota composition.
Calderón-Pérez L., et al. (Spain, 2024) [69]	134	Adults	44	Personalized nutrition diet (45) or a personalized plan (53)/Mediterranean diet (36)	21 weeks	CRP, N-acetylated proteins and MCP-1 for inflammation in order to consider different components of the inflammatory process, such as the clinical gold standard, a composite biomarker and an adipose tissue-derived inflammatory signal, respectively; and TMA for microbiota.
Chambers, E., et al. (United Kingdom, 2019) [49]	12	Non-diabetic adults with overweight and obesity	60 ± 1	Inulin-propionate ester (12) and inulin supplementation (12)/Low-fermentable fiber control-cellulose (12)	42 days (all participants completed three intervention periods)	Both IPE and inulin enhanced insulin resistance and altered gut microbiota compared to cellulose, with IPE reducing pro-inflammatory interleukin-8 levels and no significant differences between IPE and inulin.
Chashmniam, S., et al. (Iran, 2019) [50]	45	Participants with non-alcoholic fatty liver disease	Intervention: 46.56 ± 2.25 Control: 37.75 ± 3.22	Curcumin–phospholipid complex supplementation (25)/Placebo (20)	8 weeks	Decrease in oxidative and inflammatory mediators was reported.
Cofán, M., et al. (Spain and United States, 2024) [70]	115	Healthy elderly participants	Intervention: 67.9 ± 3.1 Control: 68. ± 43.0	Walnut-Enriched diet (64)/Usual diet with abstention from walnuts and avoiding other nuts (51)	24 weeks	Walnut diet increased C18:3n-3 and its oxylipins, while reducing C20:4n-6-derived metabolites, indicating a favorable shift in lipid mediator profiles.
Connolly EL, et al. (Australia, 2024) [71]	18	Participants with mild to moderately elevated blood pressure	68	Cruciferous vegetables servings added to diet (18)/Root and squash vegetables (18)	2 weeks (CI)	Active intervention increased S-methyl cysteine sulfoxide and reduced carotenoids, with associated reductions in daytime systolic blood pressure and serum triglycerides.
Corsetto, P., et al. (Italy, 2019) [51]	130	Sarcopenic elderly subjects	Intervention: 80.77 ± 6.29 Control: 80.1 ± 8.54	Liquid essential amino acids, whey protein and vitamin D supplementation combined with physical activity and a standard diet program (69)/Placebo (61)	30 days	Diet and physical activity altered fatty acid profiles, increasing dihomo-gamma-linolenic acid. Supplementation further modulated omega-6 and SFAs, suggesting a metabolic shift in the sarcopenic elderly.
Esser, D., et al. (Netherlands, 2021) [63]	19	Overweight subjects with an impaired glucose tolerance	64.2 ± 4.5	Mohana Choorna (ayurvedic herbal preparation) supplementation (19)/Placebo (19)	4 weeks (CI)	The herbal intervention increased inflammation-related gene expression but showed no correlation between urinary plant metabolites and health markers in metabolomic analysis.
Estévez-Santiago, R., et al. (Spain, 2019) [52]	72	Postmenopausal women	59 ± 6	Anthocyanins (23), Xanthophylls and Anthocyanins (26) combined with xanthophylls supplementation (23)/Between groups	8 months	Treatments had no significant impact on blood pressure or inflammation markers but improved plasma glucose (A + X), altered metabolomic profiles, and increased antioxidant capacity in all groups.
Fernández-Arroyo, S., et al. (Netherlands, 2019) [53]	24	Obese, pre-menopausal, vitamin D-deficient	Low-dose supplementation: 29 ± 3; High dose supplementation: 27 ± 2	Low dose vitamin D3 supplementation (12) and high dose vitamin D3 supplementation (12)/Between groups	7 days	Vitamin D3 supplementation in obese, vitamin D-deficient women reduced plasmatic sphingomyelin levels, highlighting its impact on lipid metabolism.
Huang, L., et al. (United States, 2021) [64]	34	Adults/subjects with moderate hypercholesterolemia	53 ± 1	Strawberry adding supplementation (34)/Control beverage consumption (34)	4 weeks (CI)	Strawberry intake increased specific phenolic metabolites and reduced 3-(4-methoxyphenyl)propanoic acid-3-O-glucuronide, which correlated with improved FMD.
Kim, C.S., et al. (Korea; 2023) [68]	53	Healthy elderly	Intervention: 71.11 Control: 72	*Bifidobacterium bifidum* BGN4 and *Bifidobacterium longum* BORI probiotics supplementation (27)/Placebo-soy oil (26)	12 weeks	Probiotics increased gut-derived IPA, a tryptophan metabolite linked to higher serum brain-derived neurotrophic factor and reduced TNFα in vitro, indicating neuroprotective and anti-inflammatory potential.
Kwon, Y.J., et al. J. et al. (Republic of Korea, 2020) [59]	68	Postmenopausal women with hypercholesterolemia	Intervention: 55.9 ± 5.9 Control: 58.1 ± 4.7	Korean red ginseng supplementation (36)/Placebo (32)	4 weeks	Korean red ginseng reduced serum cholesterol and 7-hydroxycholesterol levels, indicating improved sterol metabolism in postmenopausal women with hypercholesterolemia.
Lamon-Fava, S., et al. (United States, 2021) [65]	42	Overweight or obese adults with depressive symptomatology and chronic inflammation	Intervention: 52 ± 13, 43 ± 17 and 47 ± 15 Control: 46 ± 14	1 g/d (12), 2 g/d (11) and 4 g/d (10) of eicosapentaenoic acid supplementation/Placebo-soy oil (8)	12 weeks	EPA supplementation increased plasma EPA-derived metabolites (18-HEPE, RvE2, RvE3) dose-dependently and reduced arachidonic acid, while increasing AA-derived LXB4 at the highest dose.
Lindqvist, H., et al. (Sweden, 2019) [54]	23	Women with established rheumatoid arthritis and a disease activity score 28 > 3.0.	55	A meal including 75 g of blue mussel (*Mytilus edulis*) per day (23)/A meal including 75 g of meat per day (23)	11 weeks (CI)	Blue mussel intake altered erythrocyte fatty acid profile, increasing EPA and DHA; plasma metabolites showed no change. Erythrocyte lipids may better reflect seafood intake than plasma markers.
Liu, A., et al. (China, 2021) [66]	55	Asthmatic patients	Intervention: 54.62 ± 9.61 Control: 57.08 ± 10.46	*Bifidobacterium lactis* Probio-M8 powder and Symbicort Turbuhaler supplementation (29)/Placebo and Symbicort Turbuhaler (26)	3 months	Probiotic co-administration improved asthma control, reduced nitric oxide levels, and increased gut microbiome resilience, with enhanced beneficial metabolites and microbial diversity.
Macnaughtan, J., et al. (United Kingdom, 2020) [60]	87	Patients with cirrhosis	57.15 ± 8.83	Probiotic *Lactobacillus casei Shirota* supplementation (44)/Placebo (43)	6 months	*Lactobacillus casei Shirota* reduced inflammatory cytokines, but had no significant effect on metabolomic profile, intestinal permeability, or infection-related outcomes.
Paul, S., et al. (Australia, 2021) [67]	10	Overweight or obese males with no signs of cardiovascular disease or diabetes, plasma	50 ± 10	4 g Alkyrol^®^-purified shark liver oil supplementation (10)/Placebo-methylcellulose (10)	3 weeks (CI)	Shark liver oil supplementation increased plasmalogens and ether lipids, while reducing free cholesterol, triglycerides, and CRP levels in overweight/obese males.
Remie, C., et al. (Netherlands, 2020) [61]	13	Healthy overweight or obese participants	59 ± 5	1000 mg/d nicotinamide riboside supplementation (13)/Placebo (13)	6 weeks (CI)	Nicotinamide riboside supplementation increased NAD^+^ synthesis markers and acetylcarnitine in skeletal muscle, without affecting inflammation or mitochondrial function.
Roager, H., et al. (Denmark, 2019) [58]	50	Adults at risk of developing metabolic syndrome	48.6 ± 11.1	Whole grain intake of 179 ± 50 g/day (50)/Whole grain intake of 13 ± 10 g/day and refined grain (50)	8 weeks (CI)	Whole grain intake reduced body weight, IL-6, and CRP, linked to lower energy intake and rye consumption, but had no significant effects on glucose homeostasis or the gut microbiome.
Santos, C., et al. (Portugal, 2024) [72]	36	Adults with obesity-related hypertension and vitamin D deficiency	51.1 ± 5.5	Cholecalciferol supplementation (9)/Non cholecalciferol supplementation (9)	24 weeks	Cholecalciferol supplementation increased glutamine and histidine, decreased glucose, acetate, and altered fatty acid saturation, reflecting systemic metabolomic shifts in hypertensive patients.
Singh, D., et al. (Korea, 2024) [73]	48	Korean adults aged 25–65 years with BMI ≥ 23 kg/m2 and blood LDL cholesterol ≥ 120 mg/dL.	41	Balanced Korean diet (48), Diet recommended by the 2010 dietary guidelines for Americans (48) and Typical American diet (48)	4 weeks (CI)	Recommended diets increased urinary benzoic acid and phenolic derivatives, while Western diets showed higher oxidative stress-related metabolites; urine profiles differed clearly by diet.
Tuomainen, M., et al. (Finland, Sweden, Denmark and Iceland, 2019) [55]	163	Individuals with impaired glucose metabolism	Intervention: 54.87 ± 8.08 Control: 54.50 ± 8.70	Healthy Nordic diet-increased intakes of whole grains, canola oil, berries, and fish (70)/Control diet (93)	18/24 weeks	Pipecolic acid betaine levels increased with a healthy Nordic diet, correlating with fiber and rye intake, and inversely associated with insulin, IL-1RA, and LDL/HDL ratio.
Ulven, S., et al. (Norway, 2019) [56]	99	Healthy subjects with moderate hypercholesterolemia	55.2 ± 9.8	Replacing SFAs with PUFAs in diet (47)/Control diet (52)	8 weeks	Replacing SFAs with PUFAs reduced lipoproteins, carnitines, and kynurenine but increased bile acids, acetate, and inflammatory gene expression, indicating broad metabolic and genetic effects.
Wan, Y., et al. (China, 2019) [57]	217	Healthy young adults	Intervention: 23.3 ± 3.4, 23.6 ± 4.0 Control: 23.4 ± 4.1	Fat-controlled diet: low-fat diet-fat 20% energy (73), moderate-fat diet-fat 30% energy (73) and high-fat diet-fat 40% energy (71)	6 months	The lower-fat diet increased microbial α-diversity and decreased harmful metabolites, while the higher-fat diet reduced SCFAs and enriched pro-inflammatory factors.

AA: amino acid; BMI: body mass index; CI: crossover intervention; CRP: C-reactive protein; DHA: Docosahexaenoic acid; EPA: eicosapentaenoic acid; FMD: flow-mediated dilation; HDL: high-density lipoprotein; 18-HEPE: 18-hydroxyeicosapentaenoic acid; IL: interleukin; IPA: indole-3-propionic acid; IPE: inulin-propionate ester; LDL: low-density lipoprotein; LysoPC: lysophosphatidylcholine; LysoPE: lysophosphatidylethanolamine; LXB4: lipoxin B4; MCP-1: monocyte chemoattractant protein-1; NAD^+^: nicotinamide adenine dinucleotide; NAFLD: non-alcoholic fatty liver disease; PUFAs: polyunsaturated fatty acids; RvE2: Resolvin E2; SCFAs: short-chain fatty acids; SFAs: saturated fatty acids; TMA: trimethylamine.

**Table 3:** Effects of dietary factors on metabolites and inflammatory biomarkers according to the type of dietary intervention.

Dietary Intervention	Biosample	Effect on Metabolites		Effect on Inflammatory Biomarkers		Reference
		Increase	Decrease	Increase	Decrease	
**Interventions with food**						
A personalized diet designed to improve markers of carbohydrate metabolism	Urine and serum	Organic acids: glutamate [glutamic acid] *. Organic nitrogen compounds: dimethylamine. Organoheterocyclic compounds: allantoin. Sterol lipids: oxidized LDL.	Fatty acyls: oleic acid. Nucleic acids: pseudouridine. Organic acids: phenylalanine, tyrosine, glycine, betaine, lactate [lactic acids] *. Organic nitrogen compounds: choline, 8-oxo-2’-deoxyguanosine *.	TNFα and MCP-1 (compared to those who followed a Mediterranean diet)		Calderón-Pérez, 2024 [69]
A personalized diet aimed at improving biomarkers of inflammation		Fatty acyls: docosahexaenoic acid. Organic acids: leucine, phenylalanine, glycine, glutamate, valine, isoleucine and glutamine. Sterol lipids: LDL cholesterol * and oxidized LDL.	Organic nitrogen compounds: 8-oxo-2’-deoxyguanosine * and N-acetylglycoproteins. Glycerophospholipids: lysophosphatidylcholines *.	No effects		
A personalized diet aimed at improving biomarkers of oxidative stress		Organic acids: leucine, glutamine, methionine Organoheterocyclic compounds: allantoin	Fatty acyls: oleic acid, linoleic acid, 8-iso-PGF2α [ent-8-iso-PGF2alpha] Nucleic acids: pseudouridine. Organic nitrogen compounds: dimethylamine, trimethylamine-n-oxide	TNFα and MCP-1 (compared to those who followed a Mediterranean diet)		
A personalized diet designed to enhance biomarkers related to microbiota metabolism.		Fatty acyls: docosahexaenoic acid. Organic acids: leucine, tyrosine, glycine, glutamate, valine, isoleucine, glutamine.	Organic nitrogen compounds: 8-Oxo-2’-deoxyguanosine *, N-acetylglycoproteins, trimethylamine and dimethylamine. Glycerophospholipids: lysophosphatidylcholines *.	No effects		
A personalized diet designed to enhance lipid metabolism biomarkers		Fatty acyls: PUFAs (total) *. Organic acids: leucine, glycine, valine, glutamine. Organic nitrogen compounds: leptin *. Organoheterocyclic compounds: allantoin.	Organic nitrogen compounds: adiponectin *, 8-Oxo-2’-deoxyguanosine *.		CRP and TNFα (compared to those who followed a Mediterranean diet)	
A balanced Korean diet	Urine	Benzenoids: vanillic acid 4-o-sulfate [vanillic acid 4-sulfate] and hippuric acid. Fatty acyls: isobutyryl carnitine [CAR 3:0;2Me], cis-5-tetradecenoylcarnitine [CAR 14:1] and myristoylcarnitine [CAR 14:0]. Lignans: enterodiol-glucuronide [enterodiol] and enterolactone 3’-glucuronide *. Sphingolipids: n,n-dimethyl-safingol. Sterol lipids: 11-β-hydroxyandrosterone-3-glucuronide [11beta-hydroxyandrosterone-3-glucuronide].	Fatty acyls: oleamide and ethyl 7-epi-12-hydroxyjasmonate glucoside *. Lignans: argenteane *. Organic acids: l-isoleucyl-l-proline [ile-pro]. Organoheterocyclic compounds: creatinine. Sterol lipids: cortolone-3-glucuronide.		MCP-1	Singh, 2024 [73]
Recommended diet according to the 2010 Dietary Guidelines for Americans		Benzenoids: vanillic acid 4-O-sulfate [vanillic acid 4-sulfate], hippuric acid. Fatty acyls: isobutyryl carnitine [CAR 3:0;2Me], myristoylcarnitine [CAR 14:0], ethyl 7-epi-12-hydroxyjasmonate glucoside and 8-hydroxyfalcarinone *. Lignans: argenteane *. Organic acids: l-isoleucyl-l-proline [ile-pro]. Organoheterocyclic compounds: creatinine. Sterol lipids: 11-β-hydroxyandrosterone-3-glucuronide [11beta-hydroxyandrosterone-3-glucuronide] and 11-oxo-androsterone glucuronide.	Fatty acyls: cis-5-tetradecenoylcarnitine [CAR 14:1] and linoleamide. Lignans: enterodiol-glucuronide [enterodiol] and enterolactone 3’-glucuronide *.		MCP-1 and IL-6	
Typical American Diet		Benzenoids: hippuric acid. Fatty acyls: isobutyryl carnitine [CAR 3:0;2Me], ethyl 7-epi-12-hydroxyjasmonate glucoside, 8-Hydroxyfalcarinone *. Organic acids: l-isoleucyl-l-proline [ile-pro] and phenylacetylglutamine [alpha-n-phenylacetylglutamine]. Organoheterocyclic compounds: creatinine. Sterol lipids: 11-β-hydroxyandrosterone-3-glucuronide [11beta-hydroxyandrosterone-3-glucuronide], cortolone-3-glucuronide, 11-oxo-androsterone glucuronide.	Fatty acyls: cis-5-tetradecenoylcarnitine [CAR 14:1]. Lignans: enterodiol-glucuronide [enterodiol], enterolactone 3’-glucuronide * and argenteane*.		MCP-1	
Healthy Nordic Diet	Plasma	Alkaloids: trigonelline Organic acids: pipecolic acid betaine [(s)-homostachydrine] Organic nitrogen compounds: trimethylamine-n-oxide		No effect of pipecolic acid betaine and trigonelline on IL-1		Tuomainen, 2019 [55]
Walnut-Enriched diet	Serum	Fatty acyls: 14,15-dihydroxy-eicosatrienoic acid [14,15-DiHETrE]	Fatty acyls: 5,6-dihydroxy-epoxyeicosatrienoic acid [5,6-DiHETrE], 9-HOTrE, 13-HOTrE, α-12(13)-EpODE, 14,15-DiHETrE and 5-HETE [5S-HETE]	Not assessed		Cofán, 2024 [70]
Cruciferous vegetables servings added to diet	Urine	Organic acids: s-methyl cysteine sulfoxide		No effect on IL-6 and CRP		Connolly, 2024 [71]
	Plasma		Organic acids: s-methyl cysteine sulfoxide Organosulfur compounds: sulforaphane			
	Serum		Prenol lipids: lutein, lycopene, α-carotene β-carotene and total carotenoids			
Whole grain-rich diet	Urine	Benzenoids: pyrocatechol-glucuronide [pyrocalechol] *. Fatty acyls: 3-methyladipic acid. Organic acids: 2-aminophenol-sulfate, [2-aminophenyl sulfate] *. Organosulfur compounds: pyrocatechol-sulfate *. Phenylpropanoids and polyketides: DHPPA-glucuronide [3-(3,5-dihydroxyphenyl)propionic acid] *.			IL-6, IL-1β and TNFα	Roager, 2019 [58]
Replacing SFAs with PUFAs in diet	Plasma	Fatty acyls: acetate [acetic acid] Nucleic acids: thiamine Organic acids: serine, cystathionine, proline, citrate and asparagine Sterol lipids: bile acids	Fatty acyls: lipoprotein (XXL-VLDL, XL-VLDL, L-VLDL, M-VLDL, S-VLDL, XS-VLDL, IDL, L-LDL, M-LDL, S-LDL, XL-HDL), cholesterol (total-C, esterified-C, free-C, remnant-C, VLDL-C, LDL-C, HDL3-C), myristoylcarnitine, palmitoylcarnitine [CAR 16:0] and triglycerides (total-TG, VLDL-TG, LDL-TG, HDL-TG) Glycerophospholipids: phospholipids (total-PG, total-chol., PCs, SMs). Organic acids: cystine and kynurenine	Genes involved in inflammation		Ulven, 2019 [56]
Fat-controlled diet: Low-fat diet (fat 20% energy)	Feces	Alkaloids: 3-indolepropionic acid Fatty acyls: butyric acid	Alkaloids: indole Benzenoids: p-cresol		CRP, Thromboxane B2 and prostaglandin E2 (in low and moderate-fat diet compared to high-fat diet) Leukotriene B4 (in low and high-fat diet compared to moderate- fat diet)	Wan, 2019 [57]
Fat-controlled diet: Moderate-fat diet (fat 30% energy)		No effects.				
Fat-controlled diet: High-fat diet (fat 40% energy)		Alkaloids: indole and indoleacetic acid Fatty acyls: stearic acid, palmitic acid and arachidonic acid	Fatty acyls: butyric acid, valeric acid, ethylmethylacetic acid [2-methylbutyric acid]			
Supplementation						
Mastiha supplementation	Plasma	Sterol lipids: triterpenic acid. Sulfur inorganic compounds: sulfate metabolites.		Not assessed		Amerikanou, 2021 [62]
Inulin-propionate ester supplementation	Plasma	Fatty acyls: propionate [propionic acid]		IgG (compared to inulin and cellulose)	IL-8 (compared to inulin and cellulose)	Chambers, 2019 [49]
Inulin supplementation		No association		No effects		
Curcumin–phospholipid complex supplementation	Serum		Alkaloids: indoxyl sulfate Benzenoids: hippuric acid Organic acids: kynurenine, citric acid, 3-methyl-2-oxovaleric acid, 3-hydroxyisobutyrate acid, cuccinic acid and 2-ketoglutaric acid [2-oxoglutaric acid] Organic nitrogen compounds: trimethylamine and methylamine Sterol lipids: chenodeoxycholic acid, taurocholic acid and lithocholic acid	Not assessed		Chashmniam, 2019 [50]
Liquid essential amino acids, whey protein and vitamin D supplementation combined with physical activity and a standard diet program	Plasma	Fatty acyls: oleic acid, palmitic acid, stearic acid and SFAs Organic nitrogen compounds: glutathione reductase	Fatty acyls: linoleic acid and omega-6 PUFAs	ESR and CRP (due to palmitic, stearic and gamma-linolenic acids)		Corsetto, 2019 [51]
Mohana Choorna (ayurvedic herbal preparation) supplementation	Urine	No association		Upregulated gene sets involved in inflammation signaling pathways and immune function		Esser, 2021 [63]
Anthocyanins supplementation	Plasma	Organic acids: citric acid Organic oxygen compounds: 5-hydroxy-6-methoxyindole glucuronide	Organic nitrogen compounds: N-acetyl-L-leucine	No effects on IL-6 or other markers of inflammation assessed		Estévez-Santiago, 2019 [52]
Xanthophylls supplementation		Fatty acyls: dodecanedioic acid	Organic nitrogen compounds: N-acetyleucine			
Anthocyanins combined with xanthophylls supplementation		Benzenoids: 3,4-dimethoxybenzoic acid, 4-hydroxyhippuric acid and phenol sulfate [phenyl hydrogen sulfate]	Organic nitrogen compounds: n-acetyleucine			
Low dose vitamin D3 supplementation	Plasma	Glycerophospholipids: phosphatidylcholine 36:5	Glycerophospholipids: phosphatidylcholine (32:1 e, 32:2 e, 34:1 e, 36:2 e, 36:3 e, 36:4 e, 36:4, 36:5 e, 38:3 e, 38:4 e, 38:5 e, 38:6 e, 40:5 e, 42:4 e, 42:5 e) and phosphatidylethanolamine (38:5 e, 38:6 e) Glycerolipids: diglycerol 40:4, sphingomyelin [d18:0/16:1(9Z)]; sphingomyelin species: 32:0, 34:1, 34:2, 36:0, 36:1, 36:2, 38:1, 38:2, 40:0, 40:1, 40:2, 41:1, 42:1, 42:2, 42:3, 43:1, 43:2	Not assessed		Fernández-Arroyo, 2019 [53]
High dose vitamin D3 supplementation		Glycerophospholipids: phosphatidylethanolamine 36:4 *. Glycerolipids: diglycerol 40:4 *. Organic acids: phosphatidylcholine: 30:0, 32:0, 34:1e, 34:1, 34:2 e, 34:3, 36:1, 36:2 e, 36:2, 36:5, 38:3 e, 38:4, 38:6 e, 40:4 e, 40:6 *; Sphingolipids: sphingomyelin [d18:0/16:1(9Z)] 32:1; 33:1; 34:2 y 43:1.	Glycerolipids: diglycerol 36:3 * and triglyceride 52:2 *. Organic acids: phosphatidylcholine (38:2 and 38:4e) *.			
Strawberry adding supplementation	Plasma	Benzenoids: 4-Methoxybenzoic acid-3-sulfate. Organic acids: 3-Methoxyphenylacetic acid and 4-Hydroxyphenylacetic acid. Organosulfur compounds: hydroxybenzoic acid-sulfate *. Phenylpropanoids and polyketides: urolithin A.		No effects on apo B, apo A, and CRP		Huang, 2021 [64]
Korean red ginseng supplementation	Serum		Sterol lipids: cholesterol	Not assessed		Kwong, 2020 [59]
1 g/day EPA supplementation	Plasma	Fatty acyls: PUFA 20:5 (n-3), 22:5 (n-3) and 22:6 (n-3)	Fatty acyls: PUFA 22:5 (n-6) and 18:4 (n-3)			Lamon-Fava, 2020 [65]
2 g/day EPA supplementation		Fatty acyls: PUFA 20:5 (n-3), 22:5 (n-3) and 22:6 (n-3)	Fatty acyls: PUFA 20:2 (n-6)			
4 g/day EPA supplementation		Fatty acyls: PUFA 20:5 (n-3), 22:5 (n-3) and 22:6 (n-3)	Fatty acyls: PUFA 20:2 (n-6)		AA-derived LXB4	
Blue mussel (*Mytilus edulis*) supplementation	Serum		Carbohydrates: glucose. Organic acids: proline + unidentified metabolite in peak	Not assessed.		Lindqvist, 2019 [54]
Shark liver oil supplementation	Plasma	Glycerophospholipids: Lysoalkylphosphatidylcholine *, alkyl phosphatidylethanolamine * and lysophosphatidylethanolamine *. Organic acids: akyl phosphatidylcholine * and monoalkyldiacylglycerol *.	Glycerophospholipids: phosphatidylethanolamine *, phosphatidylinositol * and lysophosphatidylinositol *. Glycerolipids: diacylglycerol and triacylglycerol. Organic acids: phosphatidylcholine *. Sphingolipids: sphingomyelin [d18:0/16:1(9Z)]), ceramide *. Sterol lipids: cholesterol, cholesteryl ester *.		CRP	Paul, 2021 [67]
Nicotinamide riboside supplementation	Plasma		Organic nitrogen compounds: acetylcarnitine	Not associated with TNFα, IL-1α, IL-4, IL-12p70, IL-17α, CXCL10, CCL2, CCL3 or CCL4.		Remie, 2020 [61]
Cholecalciferol supplementation	Serum	Fatty acyls: lipids-CH2 and lipids-CH=CHl Organic acids: glutamine, histidine Organoheterocyclic compounds: creatinine	Carbohydrates: glucose. Fatty acyls: acetate [acetic acid] Organic acids: creatine and isoleucine	Not assessed		Santos, 2024 [77]
Interventions with probiotics						
*Bifidobacterium bifidum* BGN4 and *Bifidobacterium longum* BORI probiotics supplementation	Serum	Alkaloids: indole-3-propionic acid [3-Indolepropionic acid] and indole-3-lactic acid Organic acids: kynurenine Sterol lipids: chenodeoxycholic acid, deoxycholic acid, taurodeoxycholic acid and ursodeoxycholic acid	Alkaloids: indole-3-acetic acid [indoleacetic acid]	Indole-3-propionic acid was associated with lower levels of IL-1β and TNFα compared with lipopolysaccharide treatment		Kim, 2023 [74]
*Bifidobacterium lactis* Probio-M8 powder and Symbicort Turbuhaler supplementation	Serum	Benzenoids: syringic acid. Fatty acyls: 5-dodecenoic acid. Glycerolipids: 1-Palmitoylrac-glycerol *. Lignans: enterodiol. Organic acids: succinic acid and l-tryptophan [tryptophan]. Sphingolipids: sphingomyelin [d18:0/16:1(9Z)]. Phenylpropanoids and polyketides:1178-24-1 (3-Methoxynobiletin) *.	Fatty acyls: tetracosanoic acid [lignoceric acid]. Organic nitrogen compounds: 3-methylglutarylcarnitine. Phenylpropanoids and polyketides: schisanhenol *.	Not assessed		Liu, 2021 [66]
Probiotic *Lactobacillus casei Shirota* supplementation	Urine	No association			MCP-1	Macnaughtan, 2020 [60]
				No significant differences in plasma IL-1β, IL-2, IL-4, IL-6, IL-8, IL-10, IL-1 p70, IL-17A, IFN, MIP-1β, CRP and TNFα concentrations		

* Metabolites classified in HMDB; AA = amino acids; CAR = chimeric antigen receptor; CCL (monocyte chemoattractant protein); CPR = C-reactive protein; DHPPA: dihydroxyphenylpropionic acid; CXCL = chemokine ligand; DiHETrE = dihydroxy eicosatrienoic; EpODE: epoxy-octadecadienoic acid; HETE: hydroxyeicosatetraenoic acid; ESR = erythrocyte sedimentation rate; HOTrE: hydroxy-octadecatrienoic acid; IgG = immunoglobulin G; IL = interleukin; LDL = low density lipoprotein; MCP-1 = monocyte chemoattractant protein-1; PGF2α = prostaglandin F2 alpha; PUFA = polyunsaturated fatty acids; SFAs = saturated fatty acids; SMs = sphingomyelins; TNFα = tumor necrosis factor-alpha. Name inside brackets [ ] indicate the synonym with which each metabolite was found in the corresponding database.

## Data Availability

No new data were created or analyzed in this study.

## Supplementary Materials

The following supporting information can be downloaded at: https://www.mdpi.com/article/10.3390/metabo15110705/s1, Table S1: PRISMA 2020 Checklist; Table S2: Main characteristics of the included studies—Complete table; Table S3: Analytical approaches of the included studies.

## Abbreviations

The following abbreviations are used in this manuscript:

CRP	C-reactive protein
GC	Gas chromatography
IL	Interleukin
LC	Liquid chromatography
MS	Mass spectrometry
MCP-1	Monocyte chemoattractant protein-1
MUFAs	Monounsaturated fatty acids
NAFLD	Non-alcoholic fatty liver disease
NMS	Nuclear magnetic resonance
ox-LDL	Oxidized low-density lipoprotein
PUFAs	Polyunsaturated fatty acids
RCTs	Randomized controlled trials
SCFAs	Short-chain fatty acids
SFAs	Saturated fatty acids
TNFα	Tumor necrosis factor-alpha
