# Supplementary material for: Harnessing Metabolomics to Advance Nutrition-Based Therapeutics for Inflammation: A Systematic Review of Randomized Clinical Trials
Source: Metabolites. 2025 Oct 29;15(11):705. doi: 10.3390/metabo15110705 (PMC12654429; doi:10.3390/metabo15110705)
Supplement: Supplementary file 1 [file metabolites-15-00705-s001.zip › Supplementary material_S1.pdf]

PRISMA 2020 Checklist

Section and Topic	Item #	Checklist item	Location where item is reported
**TITLE**			
Title	1	Identify the report as a systematic review.	P1, "Harnessing Metabolomics to Advance Nutrition-Based Natural 2 Therapeutics for Inflammation: A Systematic Review of 3 Randomized Clinical Trials"
**ABSTRACT**			
Abstract	2	See the PRISMA 2020 for Abstracts checklist.	P1, "Harnessing Metabolomics to Advance Nutrition-Based Natural Therapeutics for Inflammation: A Systematic Review of Randomized Clinical Trials Background/Objectives: The association between plasma metabolites derived from dietary substrates and inflammatory processes remains underexplored, despite its potential relevance in the prevention of non-communicable diseases. This systematic review aimed to examine the relationship between blood metabolites and the modulation of inflammatory biomarkers. Methods: A total of 25 randomized controlled trials, published between 2019 and 2024, were included from an initial pool of 111 records. These studies investigated the effects of dietary patterns, specific food groups, or nutritional supplements on the human metabolome and their potential links to inflammation. Results: Metabolomic analyses were predominantly performed using MS-based platforms (17 out of 25), with LC-MS as the most frequently employed method. Both targeted (n = 14) and untargeted (n = 11) approaches were represented, and samples were drawn from plasma, urine, and feces. Across the interventions, 64 metabolites were modulated, including fatty acyls, glycerolipids, benzenoids, and organic acids, reflecting potential changes in pathways related to oxidative stress, lipid and carbohydrate metabolism, and inflammatory signaling. Several studies also assessed classical inflammatory biomarkers such as CRP, TNFα, IL-6, and MCP-1. Interventions involving healthy traditional dietary patterns, improvements in dietary fat quality, or the use of specific probiotic strains were often associated with favorable immunometabolic outcomes. In contrast, some interventions, such as Mohana Choorna, elicited upregulation of immune-related gene expression in adipose tissue without improvements in glucose or lipid metabolism. Conclusions: While metabolomic responses varied across studies, the evidence highlights the value of dietary interventions in modulating systemic metabolism and inflammation. These findings support the integration of metabolomics into clinical nutrition to define more personalized and effective dietary strategies for inflammation-related chronic disease prevention".
**INTRODUCTION**			
Rationale	3	Describe the rationale for the review in the context of existing knowledge.	Pp2-3, "A common underlying factor in the development of these diseases is chronic low-grade inflammation [33], characterized by persistently elevated levels of pro-inflammatory mediators and circulating immune cells [34–36]. Although this inflammatory state may not immediately damage tissues, it contributes significantly to disease progression [37]. Metabolomic analyses have identified specific molecules that could be involved in inflammation. These include metabolites that are significantly associated with both inflammatory and anti-inflammatory processes. This highlights their potential for use in prevention and therapeutic strategies [38]. Among the key factors influencing metabolism and the production of inflammation-modulating metabolites, diet plays a pivotal role [39]. Nutritional

Section and Topic	Item #	Checklist item	Location where item is reported
			intake shapes both host and microbiota-derived metabolites, and diet-derived metabolites generated by the gut microbiota substantially affect host metabolism [40]. Diets high in saturated fatty acids (SFAs), for example, are associated with decreased microbial diversity and increased proliferation of pathogenic species [41]. This microbial imbalance damages the gut barrier, facilitating the translocation of bacterial components into the bloodstream, triggering systemic inflammation and promoting insulin resistance. In contrast, several diet-derived metabolites exert anti-inflammatory effects [42]. For instance, short-chain fatty acids (SCFAs), such as butyrate, produced by microbial fermentation of dietary fiber, have been shown to reduce inflammation, strengthen the gut barrier, and positively modulate the immune response [43–45]”.
Objectives	4	Provide an explicit statement of the objective(s) or question(s) the review addresses.	P3, “This systematic review aims to examine the impact of different dietary substrates on metabolomic signatures and how effects on metabolism modulate inflammatory responses”.
**METHODS**			
Eligibility criteria	5	Specify the inclusion and exclusion criteria for the review and how studies were grouped for the syntheses.	P4, “A common underlying factor in the development of these diseases is chronic low-grade inflammation [33], characterized by persistently elevated levels of pro-inflammatory mediators and circulating immune cells [34–36]. Although this inflammatory state may not immediately damage tissues, it contributes significantly to disease progression [37]. Metabolomic analyses have identified specific molecules that could be involved in inflammation. These include metabolites that are significantly associated with both inflammatory and anti-inflammatory processes. This highlights their potential for use in prevention and therapeutic strategies [38]. Among the key factors influencing metabolism and the production of inflammation-modulating metabolites, diet plays a pivotal role [39]. Nutritional intake shapes both host and microbiota-derived metabolites, and diet-derived metabolites generated by the gut microbiota substantially affect host metabolism [40]. Diets high in saturated fatty acids (SFAs), for example, are associated with decreased microbial diversity and increased proliferation of pathogenic species [41]. This microbial imbalance damages the gut barrier, facilitating the translocation of bacterial components into the bloodstream, triggering systemic inflammation and promoting insulin resistance. In contrast, several diet-derived metabolites exert anti-inflammatory effects [42]. For instance, short-chain fatty acids (SCFAs), such as butyrate, produced by microbial fermentation of dietary fiber, have been shown to reduce inflammation, strengthen the gut barrier, and positively modulate the immune response [43–45]”.
Information sources	6	Specify all databases, registers, websites, organisations, reference lists and other sources searched or consulted to identify studies. Specify the date when each source was last searched or consulted.	P3, “This study only included original publications written in English. Reviews and meta-analyses, comments, guidelines, editorials or letters, conference summaries, and non-randomized or non-controlled studies were excluded. The inclusion and exclusion criteria are outlined in Table 1”.
Search strategy	7	Present the full search strategies for all databases, registers and websites, including any filters and limits used.	Pp3-4, “An electronic search was performed using PubMed, Cochrane Library, and Epistemonikos databases (last searched in September 2024) to identify all articles related to the role of different dietary substrates on the profiles of metabolites

PRISMA 2020 Checklist

Section and Topic	Item #	Checklist item	Location where item is reported
			present in humans and how they influence inflammatory tone. The following combination of Medical Subject Heading (MeSH) terms and text words were used: (Inflammation) AND (Nutrition OR Food OR Nutrient* OR Diet) AND (Metabolomic OR Metabolomics OR Metabolome OR Lipidomic OR Lipidomics OR Lipidome OR "Metabolic Profile" OR "Metabolic Profiles" OR "Metabolic Profiling" OR "Metabolite Profile" OR "Metabolite Profiles" OR "Metabolite Profiling")".
Selection process	8	Specify the methods used to decide whether a study met the inclusion criteria of the review, including how many reviewers screened each record and each report retrieved, whether they worked independently, and if applicable, details of automation tools used in the process.	-
Data collection process	9	Specify the methods used to collect data from reports, including how many reviewers collected data from each report, whether they worked independently, any processes for obtaining or confirming data from study investigators, and if applicable, details of automation tools used in the process.	P4, "Data was extracted in a standardized format into a Microsoft Excel® spreadsheet. The following information was collected from each included study: Title, Authors, Year of Publication, Journal, DOI, Search Query, Date of Inclusion in the Database, Filters, RCT Research, Source, Metabolomic Technique, Samples, availability of the paper Free/Not Free, and Additional Comments. Four reviewers independently assessed and selected the studies based on the inclusion, exclusion, and quality assessment criteria outlined in the Risk of Bias Tool (Rob2). Discrepancies were resolved by discussion to reach a consensus".
Data items	10a	List and define all outcomes for which data were sought. Specify whether all results that were compatible with each outcome domain in each study were sought (e.g. for all measures, time points, analyses), and if not, the methods used to decide which results to collect.	-
	10b	List and define all other variables for which data were sought (e.g. participant and intervention characteristics, funding sources). Describe any assumptions made about any missing or unclear information.	-
Study risk of bias assessment	11	Specify the methods used to assess risk of bias in the included studies, including details of the tool(s) used, how many reviewers assessed each study and whether they worked independently, and if applicable, details of automation tools used in the process.	P4, "The quality of the included studies was assessed using the Cochrane risk of bias tool for randomized trials (Rob2) (https://methods.cochrane.org/bias/resources/rob-2-revised-cochrane-risk-bias-tool-randomized-trials). The Rob2 tool allows for the assessment of bias that may arise at different stages of an RCT across five distinct domains. The five assessed domains represent: (1) Bias in the randomization process, (2) Bias due to deviation from intended interventions, (3) Bias due to missing outcome data, (4) Bias in the measurement of the outcome, (5) Bias in the selection and reporting of results. Each domain was rated as having a "low risk", "some concerns", or "high risk" of bias".
Effect measures	12	Specify for each outcome the effect measure(s) (e.g. risk ratio, mean difference) used in the synthesis or presentation of results.	-
Synthesis methods	13a	Describe the processes used to decide which studies were eligible for each synthesis (e.g. tabulating the study intervention characteristics and comparing against the planned groups for each synthesis (item #5)).	-
	13b	Describe any methods required to prepare the data for presentation or synthesis, such as handling of missing summary statistics, or data	-

Section and Topic	Item #	Checklist item	Location where item is reported
		conversions.	
	13c	Describe any methods used to tabulate or visually display results of individual studies and syntheses.	-
	13d	Describe any methods used to synthesize results and provide a rationale for the choice(s). If meta-analysis was performed, describe the model(s), method(s) to identify the presence and extent of statistical heterogeneity, and software package(s) used.	-
	13e	Describe any methods used to explore possible causes of heterogeneity among study results (e.g. subgroup analysis, meta-regression).	-
	13f	Describe any sensitivity analyses conducted to assess robustness of the synthesized results.	-
Reporting bias assessment	14	Describe any methods used to assess risk of bias due to missing results in a synthesis (arising from reporting biases).	P4, “Bias due to missing outcome data”.
Certainty assessment	15	Describe any methods used to assess certainty (or confidence) in the body of evidence for an outcome.	-
**RESULTS**			
Study selection	16a	Describe the results of the search and selection process, from the number of records identified in the search to the number of studies included in the review, ideally using a flow diagram.	P5, “The flow diagram of the study selection process is shown in Figure 1. In the original search, 111 records were published from 2019 to 2024. Before screening, 14 records were removed: 2 duplicates, 7 trial registrations, and 5 records with inaccessible full text. A total of 97 records remained and were screened by title and abstract.
	16b	Cite studies that might appear to meet the inclusion criteria, but which were excluded, and explain why they were excluded.	P5, “Of these, 34 were excluded for not meeting eligibility criteria. The remaining 63 articles were assessed in full-text form. Among those, 38 were excluded for the following reasons: inadequate outcome (n= 9), inadequate study design (n= 2), no conventional metabolomics instrument used (i.e., methods other than MS or NMR) (n= 12), not metabolomics-focused (studies centered on biomarkers rather than direct metabolite profiling) (n= 12), questionable study integrity (n= 1), and absence of a comparison group between intervention and control (n= 2). Ultimately, 25 studies met all inclusion criteria and were included in the systematic review. Ultimately, 25 studies met all inclusion criteria and were included in the systematic review. Of these, 8 were crossover RCTs and 17 were parallel-group RCTs”.
Study characteristics	17	Cite each included study and present its characteristics.	P5-6, “The main characteristics of the included studies are summarized in Table 2, while comprehensive details are provided in Supplementary Table S1. Ten studies were published in 2019 [47–56], three in 2020 [57–59], six in 2021 [60–65], one in 2023 [66], and five in 2024 [67–71]. The geographical origins of the included studies were diverse. Most were conducted in Europe, particularly in Greece [60], Italy [49,60], Serbia [60], Spain [50,67,68], United Kingdom [47,58], Netherlands [51,59,61], Denmark [53,56], Portugal [70], Finland [53], Sweden [52,53], Iceland [53], and Norway [54]. Three were conducted in the United States [62,63,68], two in Australia [65,69], and the rest in Asia: Iran [48], Korea [57,71,72], and China

Section and Topic	Item #	Checklist item	Location where item is reported
			[55,64]. Of these, three were multi-centric: two international [53,60] and one intercontinental [68]. The sample sizes ranged from 10 [65] to 217 [55], with a total of 1654 individuals across all studies. The analysis included healthy individuals as well as those diagnosed with conditions such as non-alcoholic fatty liver disease, overweight or obesity, elevated blood pressure, impaired glucose tolerance, vitamin D deficiency, hypercholesterolemia, depressive symptomatology, rheumatoid arthritis, asthma, cirrhosis, and hypertension. One study included only men [65], while four were conducted exclusively in women [50–52,57]. The remaining studies included participants of both sexes, except for one [66] that did not report on sex. The mean age of the participants ranged between 41 [71] and 81 [49] years. The duration of the intervention varied from 7 days [51] to 2 years [68]. Eight of the interventions focused on food or dietary patterns, such as the Mediterranean diet or dietary fat control. Fourteen involved supplementations, such as vitamin D and Korean red ginseng, and three involved probiotics (Table 2)".
Risk of bias in studies	18	Present assessments of risk of bias for each included study.	-
Results of individual studies	19	For all outcomes, present, for each study: (a) summary statistics for each group (where appropriate) and (b) an effect estimate and its precision (e.g. confidence/credible interval), ideally using structured tables or plots.	-
Results of syntheses	20a	For each synthesis, briefly summarise the characteristics and risk of bias among contributing studies.	-
	20b	Present results of all statistical syntheses conducted. If meta-analysis was done, present for each the summary estimate and its precision (e.g. confidence/credible interval) and measures of statistical heterogeneity. If comparing groups, describe the direction of the effect.	-
	20c	Present results of all investigations of possible causes of heterogeneity among study results.	-
	20d	Present results of all sensitivity analyses conducted to assess the robustness of the synthesized results.	-
Reporting biases	21	Present assessments of risk of bias due to missing results (arising from reporting biases) for each synthesis assessed.	-
Certainty of evidence	22	Present assessments of certainty (or confidence) in the body of evidence for each outcome assessed.	-
**DISCUSSION**			
Discussion	23a	Provide a general interpretation of the results in the context of other evidence.	Pp30-34, "Integrating targeted and untargeted metabolomics into dietary intervention research provides a rigorous framework for identifying molecular mediators underlying the health effects of specific dietary patterns and supplements [75]. Rather than relying solely on clinical endpoints, combining both approaches allows to identify bioactive compounds, host-microbiota co-metabolites, and signaling molecules that reflect real-time biological responses to

Section and Topic	Item #	Checklist item	Location where item is reported
			nutritional exposure [76]. Across the reviewed studies, diverse interventions ranging from whole dietary patterns to targeted supplementation, consistently modulated the levels of various metabolites and showed potential link to inflammatory biomarkers".
	23b	Discuss any limitations of the evidence included in the review.	P34,"Several methodological limitations were consistently observed across the included studies, which may affect the strength of the evidence synthesized in this review: (a) several trials were affected by pandemic-related constraints, such as limited in-person contact and reliance on electronic participation, which might have impacted adherence and reporting [67,69]; (b) small sample sizes, limited ethnic representation, wide age ranges, and lack of personalized or representative diets reduced generalizability [48,52,58,60,61,63,70,71]; (c) incomplete dietary controls posed risks of unreported food intake or medication use, introducing confounding factors [54,57,63,70]; (d) short intervention durations, absence of follow-up, single-blind designs, and inconsistent handling of biological samples, all potentially affecting data quality [48,57,59,61,67,68,71]; and (e) the absence of complementary analyses like biopsies or a comprehensive lipidome profiling, use of diverse metabolomic techniques, limits the understanding of the underlying mechanisms associated with the metabolomic observed metabolomics changes".
	23c	Discuss any limitations of the review processes used.	P35," This systematic review explored a broad range of dietary exposures and metabolites measured by several techniques. While the heterogeneity in terms of study population and endpoints of each RCT, makes it difficult to generalize findings between studies, some consistent patterns can still be identified".
	23d	Discuss implications of the results for practice, policy, and future research.	Pp34-35, "The findings of this review highlight the clinical potential of integrating metabolomic profiling into dietary interventions aimed at modulating inflammation. The consistent identification of metabolite patterns associated with anti-inflammatory effects, including trigonelline, carnitines, sphingolipids, and microbial-derived compounds, suggests that specific dietary strategies can induce measurable biological changes well before conventional clinical markers would detect them. This positions metabolomics as a valuable early detection tool for evaluating dietary efficacy and tailoring personalized nutrition approaches in clinical practice. The reviewed evidence supports dietary recommendations centered on whole-food-based patterns and improved fat quality, such as Mediterranean, Nordic, and low saturated fat diets, for their capacity to favorably modulate metabolic and inflammatory pathways. Clinically, this reinforces the rationale for incorporating personalized dietary advice not only to improve cardiometabolic risk factors but also to promote immune balance via nutrient-microbiota-host interactions. Even though not every intervention showed a global trend toward reducing chronic inflammation, these results highlight the importance of incorporating multiomic analyses to understand the complex interactions between diet, metabolism, and immune responses. Future research, integrating metabolomics with traditional clinical endpoints, may enhance the precision of nutritional interventions and improve the prevention and management of inflammation-related chronic diseases".

PRISMA 2020 Checklist

Section and Topic	Item #	Checklist item	Location where item is reported
**OTHER INFORMATION**			
Registration and protocol	24a	Provide registration information for the review, including register name and registration number, or state that the review was not registered.	-
	24b	Indicate where the review protocol can be accessed, or state that a protocol was not prepared.	-
	24c	Describe and explain any amendments to information provided at registration or in the protocol.	-
Support	25	Describe sources of financial or non-financial support for the review, and the role of the funders or sponsors in the review.	P35, “Funding: This work was supported by (a) Doctoral scholarship to B.C., Consejo Nacional de Investigaciones Científicas y Técnicas (CONICET), Ministry of Science and Technology, Argentina (RESOL-2021-154-APN-DIR#CONICET); (b) Doctoral scholarship to G.N.G.-F., Consejo Nacional de Investigaciones Científicas y Técnicas (CONICET), Ministry of Science and Technology, Argentina (RESOL-2018-2704-APN-DIR#CONICET)”.
Competing interests	26	Declare any competing interests of review authors.	P35, “The authors declare no conflict of interest”.
Availability of data, code and other materials	27	Report which of the following are publicly available and where they can be found: template data collection forms; data extracted from included studies; data used for all analyses; analytic code; any other materials used in the review.	-

From: Page MJ, McKenzie JE, Bossuyt PM, Boutron I, Hoffmann TC, Mulrow CD, et al. The PRISMA 2020 statement: an updated guideline for reporting systematic reviews. BMJ 2021;372:n71. doi: 10.1136/bmj.n71.
This work is licensed under CC BY 4.0. To view a copy of this license, visit https://creativecommons.org/licenses/by/4.0/
